# *CKMT1A* is a novel potential prognostic biomarker in patients with endometrial cancer

**DOI:** 10.1371/journal.pone.0262000

**Published:** 2022-01-25

**Authors:** Yaping Wang, Shujun Zhao, Qiaohong Qin, Xiang Gao, Xinlu Zhang, Min Zhang, Yi Jiang, Xiaorong Ji, Hai Zhu, Xin Zhao, Hongyu Li

**Affiliations:** 1 Gynecologic Oncology, the Third Affiliated Hospital of Zhengzhou University, Zhengzhou, Henan, China; 2 Zhengzhou Key Laboratory of gynecological oncology, Zhengzhou, Henan, China; 3 The Third Affiliated Hospital of Zhengzhou University, Zhengzhou, Henan, China; University of Calgary, CANADA

## Abstract

**Purpose:**

The International Federation of Gynecology and Obstetrics (FIGO) stage remains the standard staging system for the assessment of endometrial cancer (EC) prognosis. Thus, we aim to identify the significant genes or biomarkers associated with the stage of endometrial cancer, which may also help reveal the mechanism of EC progression and assess the prognosis of patients with EC.

**Materials and methods:**

We compared the mRNA expression levels of EC patients with stages I and II as well as stages III and IV in the Cancer Genome Atlas (TCGA) database. The differentially expressed genes (DEGs) of EC patients at different stages were selected by volcano plot and Venn analysis. Gene Ontology (GO) and Pathways were applied to analyze the identified genes. Protein protein interaction (PPI) network was employed to identify the correlation. The survival analyses based on TCGA database were conducted for further screening. The Human Protein Atlas, quantitative PCR and immunohistochemistry were utilized to confirm the differences in expression of DEGs in endometrial cancer samples at different FIGO stages.

**Results:**

*CKMT1A* was identified as a candidate gene. Through survival analyses, we found that *CKMT1A* may be a poor prognostic factor in the overall survival of endometrial cancer patients. GO and Pathways revealed that *CKMT1A* is closely associated with the metabolic process. More importantly, Human Protein Atlas and quantitative PCR confirmed the differences in expression of *CKMT1A* in endometrial cancer samples at different FIGO stages.

**Conclusion:**

In summary, this study shows that *CKMT1A* is a newly identified essential tumor progression regulator of endometrial cancer, which may give rise to novel therapeutic strategies in the management of endometrial cancer patients to prolong its prognosis and prevent tumor progression.

## Introduction

Endometrial cancer (EC) is one of the most common malignant tumors in the female reproductive tract [1]. According to the newest cancer statistics from American Cancer Society, the number of new endometrial cancer cases in the United States in 2020 approximately is 65,620 with 12,590 deaths [2]. In the past several years, the incidence of EC has increased according to the age and size of the population [3]. The majority of patients with endometrial cancer are diagnosed at an early stage (the disease is limited to the uterine corpus), which may be treated by surgery with or without adjuvant treatment [4]. Approximately 75% of early EC patients survive for 5 years. However, the prognosis of patients with advanced, recurrent, or metastatic EC remain unsatisfactory [5]. Therefore, it is critical to determine the novel molecules that may serve as prognostic markers and therapeutic targets in the management of EC.

The Cancer Genome Atlas (TCGA) database provides high-throughput sequencing and gene expression profile data for 33 types of cancer, facilitates research on genes involved in cancer development and progression, and helps identify the promising biomarkers for cancer prognosis. The data from TCGA is publicly available and has a profound effect on understanding the molecular features of cancer occurrence while improving the treatment and prognosis of the respective types of cancer [6]. Recently, many prognostic markers of EC were discovered using exhaustive genomic and transcriptome analyses of EC using TCGA gene expression profile data, significantly promoting the development of effective targets in the treatment of EC [7–9]. SRY-Box Transcription Factor 11(*SOX11*) hypomethylation is an independent poor prognostic marker in EC [10]. Similarly, the overexpression of neurotensin receptor 1 (*NTSR1*) is a poor prognostic factor in EC [7]. By analyzing the TCGA database, the high levels of expression among Kallikrein-5-8(*KLK5-8*) and Matrix metalloproteinase-20(*MMP20*) may demonstrate their roles as potential prognostic biomarkers for a poor prognosis of EC [9, 11]. Moreover, the expression of microRNA-124 was downregulated in tumor tissues and associated with poor survival among EC patients, which was considered to be a poor prognostic marker in EC [12]. Furthermore, the low levels of microRNA-449a and microRNA-145-5 were found to be associated with poor clinical outcomes in EC [13]. However, no study has directly screened mRNA profiles based on the International Federation of Gynecology and Obstetrics (FIGO) staging system. In this study, we divided EC patients into different groups based on their respective FIGO stages and screened the mRNA profiles for each group.

*CKMT1A*, creatine kinase mitochondrial 1A, also known as *U-MtCK*, *CKMT1*, or *mia-CK*, is a kind of mitochondrial creatine kinase and is part of the creatine kinase isoenzyme family. The transfer of high energy phosphates from the mitochondria to the cytosolic carrier was believed to be the function of *CKMT1A* [14]. Previous studies have reported that the over-expression of *CKMT1A* was related to acute myeloid leukemia (AML), breast cancer, hepatocellular carcinoma (HCC), neck squamous cell carcinoma and nasopharyngeal carcinoma [15–19]. However, to the best of our knowledge, the expression of *CKMT1A* in association with the prognosis of EC patients has yet to be elucidated. Therefore, our study is purposed to reveal the relationship between the expression of *CKMT1A* in EC and the prognosis of patients with EC for accurately predicting the patients’ prognosis.

To determine the candidate target genes that may prolong the survival of patients undergoing EC treatment, we compared the mRNA expression profiles of tissues derived from the two different groups of EC samples in the TCGA database based on the FIGO stage. Firstly, the mRNA expression was compared between EC patients of stages I and II from the TCGA database and then of stages III and IV. Next, a Venn analysis was performed to determine which candidate genes were up-regulated in the advanced stages of disease among patients with a poor prognosis. Furthermore, metabolism-associated pathway enrichment was carried out through Gene Ontology (GO) and gene set enrichment analysis (GSEA). Finally, the data from the human protein atlas and clinical samples was obtained to verify the clinical significance of the identified genes. Our findings may provide further insight and understanding into the development of endometrial cancer.

## Materials and methods

### RNA-seq data

We downloaded the gene expression profile data of EC from TCGA (http://cancergenome.nih.gov/), which contained RNA-seq data for 175 samples as well as 164 tumor samples and 11 adjacent normal tissues from EC patients. Herein, 90 samples at stage I, 22 samples at stage II, 42 samples at stage III and 10 samples at stage IV were collected. Any relevant clinical information was also obtained.

### Screening of differentially expressed genes (DEGs)

The data was normalized before comparison. The gene expression data of EC downloaded from the TCGA database was divided into two groups according to stage. R Language was used to investigate differentially expressed genes (DEGs) between endometrial cancer patients in stages I and II. DEGs were then obtained based on the genetic expression of stages III and IV. With a threshold P value<0.05 and a false discovery rate (FDR) of P<0.05, DEGs were identified. FDR was used to adjust the P value that controls type I errors.

### Venn analysis

The gene expression data collected from TCGA database was divided into two groups based on the FIGO stage. Firstly, stages I and II were sorted, followed by stages III and IV. The differential expressions of the genes in each tissue were shown using web tool Venn diagrams (http://bioinformatics.psb.ugent.be/webtools/Venn). Therefore, the specific genes related to the prognosis of EC patients may be identified.

### GO annotation analysis

A functional analysis of DEGs was conducted by the GO project (http://www.geneontology.org) based on biological processes [20].

### Pathway annotation analysis

Based on the Kyoto Encyclopedia of Genes and Genomes (KEGG), Reactome and BioCarta, a path analysis was performed to determine the significant pathways involving DEG.

### PPI network construction

To uncover the functional associations between proteins across the genome, we evaluated protein–protein interaction (PPI) information using an online tool, STRING [21] (https://string-db.org). We inputted the DEGs into STRING and set the confidence score ≥ 0.4 with the maximum number of interactors = 50 as a threshold. In the PPI network, each node indicates a protein, and each edge indicates an interaction involving pairwise proteins. The nodes with a comparatively large amounts of edges were determined to be hub proteins. Colored nodes represent query proteins and the first shell of interactors. Filled nodes represent some 3D structures that are known or predicted. White nodes represent second shell of interactors. The light blue is an auxiliary database evidence. The purple line is experimental proof. The yellow and yellow-green lines are the text digging evidence. The green line is similar genes. The red line is gene fusion. The blue line is co-produced by genes. The black line is gene co-expression. The gray line is the protein homology.

### Gene set enrichment analysis (GSEA)

GSEA was conducted using the GSEA 3.0 software, and the genome used in this study was downloaded from the Molecular Signatures Database (MSigDB, http://software.broadinstitute.org/gsea/msigdb/index.jsp, v4.0, released Jun 7, 2013). MSigDB curate various gene sets including 1,320 canonical pathways from BioCarta, KEGG, PID, Reactome and other pathway databases. According to the expression level of *CKMT1A*, TCGA patients were divided into high expression and low expression groups. One can download the expression matrix expression dataset (gct file, common txt format is also available) and the sample grouping information phenotype labels (cls file), and fill in the grouped data into the template file. After preparing sample expression quantity files and sample phenotype classification files, the TCGA data was analyzed in GSEA, and the pathways with an FDR <0.05 were considered to be significant.

### TCGA database analysis

The data of TCGA database was collected from the UCSC Cancer Browser (https://genome-cancer.ucsc.edu). Kaplan-Meier survival plots were used to present the overall survival (OS) of endometrial cancer patients with stage of different genes. The cut-off value for the genes was the stage. A Kaplan-Meier curve was used to show the prognosis for the high and low groups. A log-rank t test was then applied on the Kaplan-Meier curve to detect survival differences between the two groups.

### The Human Protein Atlas

Consisting of the Cell Atlas, Tissue Atlas and Pathology Atlas, the Human Protein Atlas (https://www.proteinatlas.org/) provided a large quantity of transcriptomics and proteomics data in specific tissues of the human body. The database included the information of 20 common tumor types and 44 different normal tissues and organs. Additionally, the immunohistochemistry (IHC) protein expression in tumor tissues and normal human tissues was downloaded from the Human Protein Atlas. In our study, we utilized the database to investigate the protein expression of *CKMT1A* in both normal endometrial and cancerous endometrial tissues.

### Clinical specimens

A total of 39 endometrial tumor tissues were obtained from the patients who were diagnosed and received primary surgery at the third Affiliated Hospital of Zhengzhou University (Zhengzhou, China) from 2013 to December 2015. We collected relevant clinical information and other overall survival data via medical records and telephone inquiries. All samples were collected at the time of surgery and were immediately stored in liquid nitrogen (-80°C). The diagnosis of EC was made by at least two independent pathologists. The biological samples were completely de-identified before researchers accessed the samples and the study was approved by the Ethics Committee of the Third Affiliated Hospital of Zhengzhou University (Ethical approval number: Science-2019-LW-101). Furthermore, we obtained written informed consent from each patient with accessible follow-up information.

### Quantitative real-time polymerase chain reaction (qRT-PCR)

A total of 39 endometrial tumor tissues were used in our study. The total RNA from these tissues were extracted using Trizol solution (Invitrogen, Waltham, MA, USA) according to the manual. Independently, the RNA of each sample was reverse-transcribed using the PrimeScript RT reagent kit (Takara Bio, Otsu, Shiga, Japan). qRT-PCR was performed by employing specific primers and the SYBR Green qPCR Master Mix (Takara, Japan). The specific primers used are as follows: 5’- AAAGCCTGCCGGTGACTAAC-3’ sense primer and 5’- ACATGTAAACCATGTAGTTGAGGT -3’ antisense primer for glyceraldehyde-3phosphate dehydrogenase (*GAPDH*); and 5’- CACAGGAACTAGGAACTACGGA-3’ sense primer, 5’-GCCAATCAGACTTTGATCTTTTACT-3’ antisense primer for *CKMT1A*. The expression levels of the clinical samples were compared with the 2-ΔΔCt method(22). Each sample’s expression of *CKMT1A* and *GAPDH* was detected, where *GAPDH* was regarded as an internal control. Then, the relative expression levels of *CKMT1A* were calculated using the 2-ΔCt value of *CKMT1A* divided by the 2-ΔCt value of *GAPDH* [22].

### Immunohistochemistry

A total of 40 endometrial cancer tissues and 40 endometrial paratumor tissues were involved in this study. There were ten cases each in stages I, II, III and IV, respectively. The paraffin-embedded tissues of 80 samples were examined for the expression of *CKMT1A* protein (Abcam, Cambridge, UK; 1:300). The sections were treated with 3% H2O2 and 5% bull serum albumin (BSA), prior to incubation with the primary antibody at 4°C overnight. After the incubation with horseradish peroxidase (HRP) conjugated secondary antibody at 37°C for 1 hour, the sections were washed and counterstained using hematoxylin for subsequent observation under microscope (Olympus, Shinjuku, Japan).

### Statistical analysis

Bioinformatics analyses were conducted using the R software (version 3.4; R Foundation for Statistical Computing, Vienna, Austria). The Chi-square test and Fisher Chi-square test were conducted to compare the clinicopathological factors, and the Student’s t-test and one-way Analysis of Variance (ANOVA) were performed to analyze the continuous variables. A survival analysis was conducted using a Kaplan-Meier analysis and log rank t-test. Moreover, univariate and multivariate logistic regression models were used to confirm the correlations between the expression levels of *CKMT1A* with the corresponding clinical features. The statistics related to the clinical samples were analyzed using Prism 7 (GraphPad Software Inc., La Jolla, USA). Statistical analyses were conducted using ANOVA, and a chi-square test was performed with SPSS 16.0 for Windows (SPSS Inc., Chicago, IL, USA). P <0.05 was regarded as statistically significant.

## Results

### Gene expression analysis

The gene expression data of EC downloaded from the TCGA database (https://cancergenome.nih.gov/) included 90 EC patients at stage I (I), 22 at stage II (II), 42 at stage III (III) and 10 at stage IV (IV) as well as 11 normal adjacent tissues. Detailed sample related material may be found in the S1 Table.

### Identification of specific gene signatures

**Fig 1:**
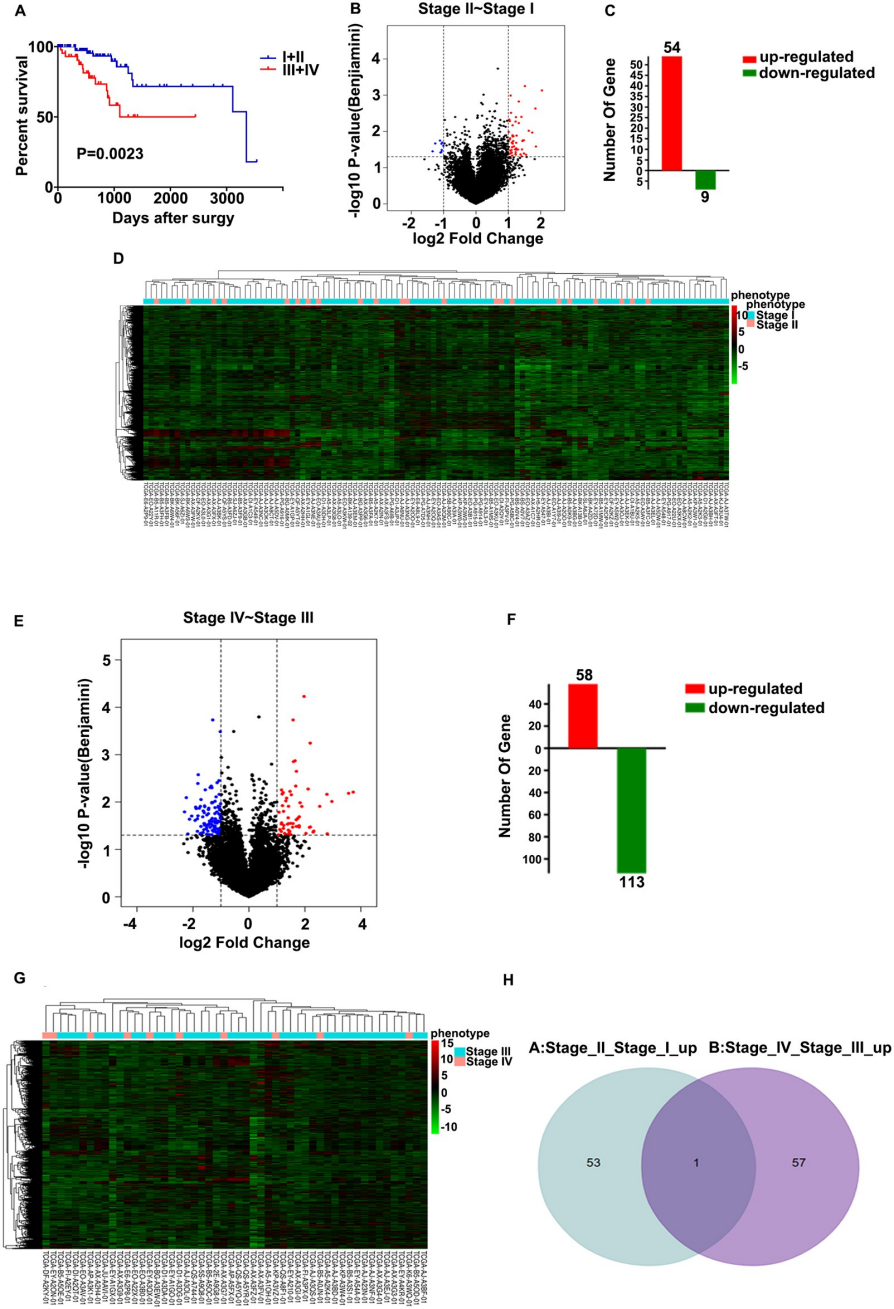
Differences in expression of genes among stages I and IV of endometrial cancer. (A) According to the expression of the mRNA levels from TCGA database, the patients were divided into 2 groups based on their respective FIGO stages. (B) The volcano plot of mRNA levels from the TCGA database and the x-axis represent the log2 transformed of fold change ratios. The y-axis is the log10 transformed adjusted p-value. The red colored dots represent the DEGs based on fold change >1. Herein, the volcano plot displayed the different genes when comparing patients in the stage II group with the patients in the stage I group. (C) Based on volcano analysis, the plot of 54 upregulated and 9 downregulated genes. (D) Heatmap of the candidate genes associated with FIGO stage from TCGA database. (E) Volcano plot of the mRNA levels of different genes in samples with stage IV and samples with stage III. (F) The plot showed 58 upregulated and 113 downregulated genes based on the above volcano analysis. (G) Heatmap of the candidate genes associated with FIGO stage from TCGA database. (H) Venn diagram representing the distribution of DEGs in different groups. 1 DEG was upregulated in both scenarios of stage IV vs. stage III and stage II vs. stage I.

To identify the DEGs between stages as well as the prognostic genes for endometrial cancer, the patients were separated into two groups according to their respective FIGO stages: stages I and II and stages III and IV (Fig 1A). To identify the critical genes associated with their stage in these patients, the mRNA expression levels in stage II vs. I and stage IV vs. III were compared using the R software. After analyzing the transcriptomic changes of stage II vs. I, a total of 63 differentially expressed genes (DEGs) were identified in the Volcano plot (Fig 1B) (S2 Table), including 54 upregulated and 9 downregulated transcription factors (Fig1C). The corresponding expression levels of the 63 obtained DEGs were shown using a Heatmap (Fig 1D). The DEGs between stages III and IV were then determined using a Volcano plot (Fig 1E), where a total of 171 DEGs were observed (S3 Table), including 58 up-regulated and 113 down-regulated transcription factors (Fig 1F). The expression of the 171 DEGs were shown in a heatmap (Fig 1G). Finally, a Venn diagram was established, showing 1 DEG according to the two screening methods (Fig 1H). The obtained results implied that the gene may play a critical role in the progression of endometrial cancer, whose levels were changed in the progression of cancer. Therefore, we selected the hub gene *CKMT1A* in order to study its association with patient prognosis for further analysis.

### Significant GOs and pathways

**Fig 2:**
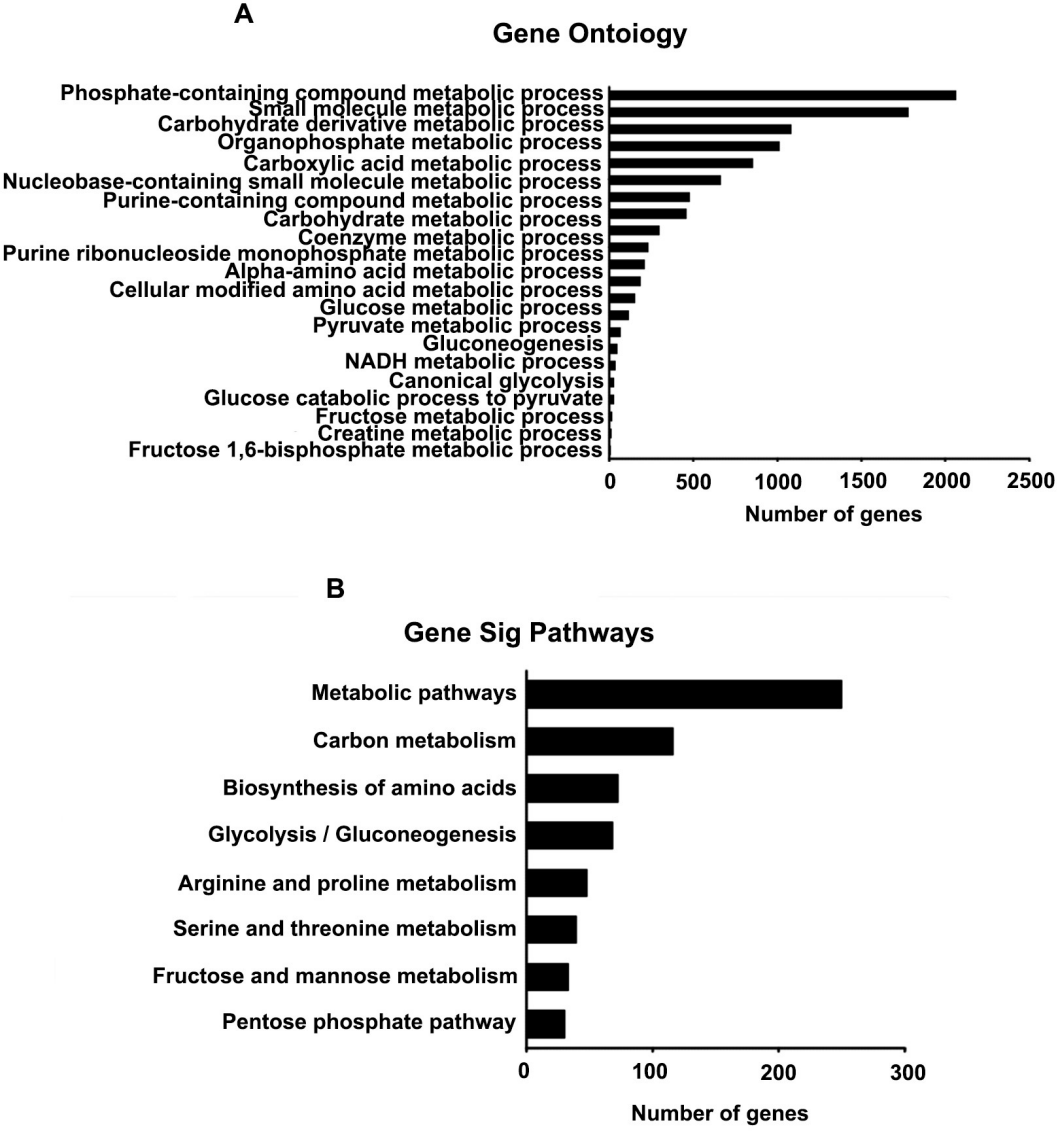
Functional analysis for CKMT1A. (A) GO analysis of CKMT1A. (B) KEGG analysis of CKMT1A.

GO annotation was used to conduct functional analyses of the identified DEGs, involving different biological processes, cellular components, and molecular functions. The results showed that *CKMT1A* mainly participated in 70 significant GOs (S4 Table; P<0.05). A plethora of metabolic processes, such as those in phosphate-containing compounds, small molecules, carbohydrate derivates, organophosphates, carboxylic acid, nucleobases with small molecules, purine-containing compounds, and carbohydrates, may be involved in the progression of endometrial cancer (Fig 2A). Therefore, it may be inferred that *CKMT1A* is mainly involved in metabolic processes.

To identify the key pathways in involving *CKMT1A* activity, a pathway analysis was performed, and 8 KEGG biological pathways were annotated (S5 Table). The major biological pathways include the biosynthesis of amino acids, metabolic pathways, carbon metabolism, glycolysis/gluconeogenesis, arginine and proline metabolism, glycine, serine and threonine metabolism, fructose and mannose metabolism, and pentose phosphate pathway. The results revealed that *CKMT1A* may be a key molecule in endometrial cancer, which is known to have a close relation with metabolic diseases (Fig 2B).

### A PPI network of genes

**Fig 3:**
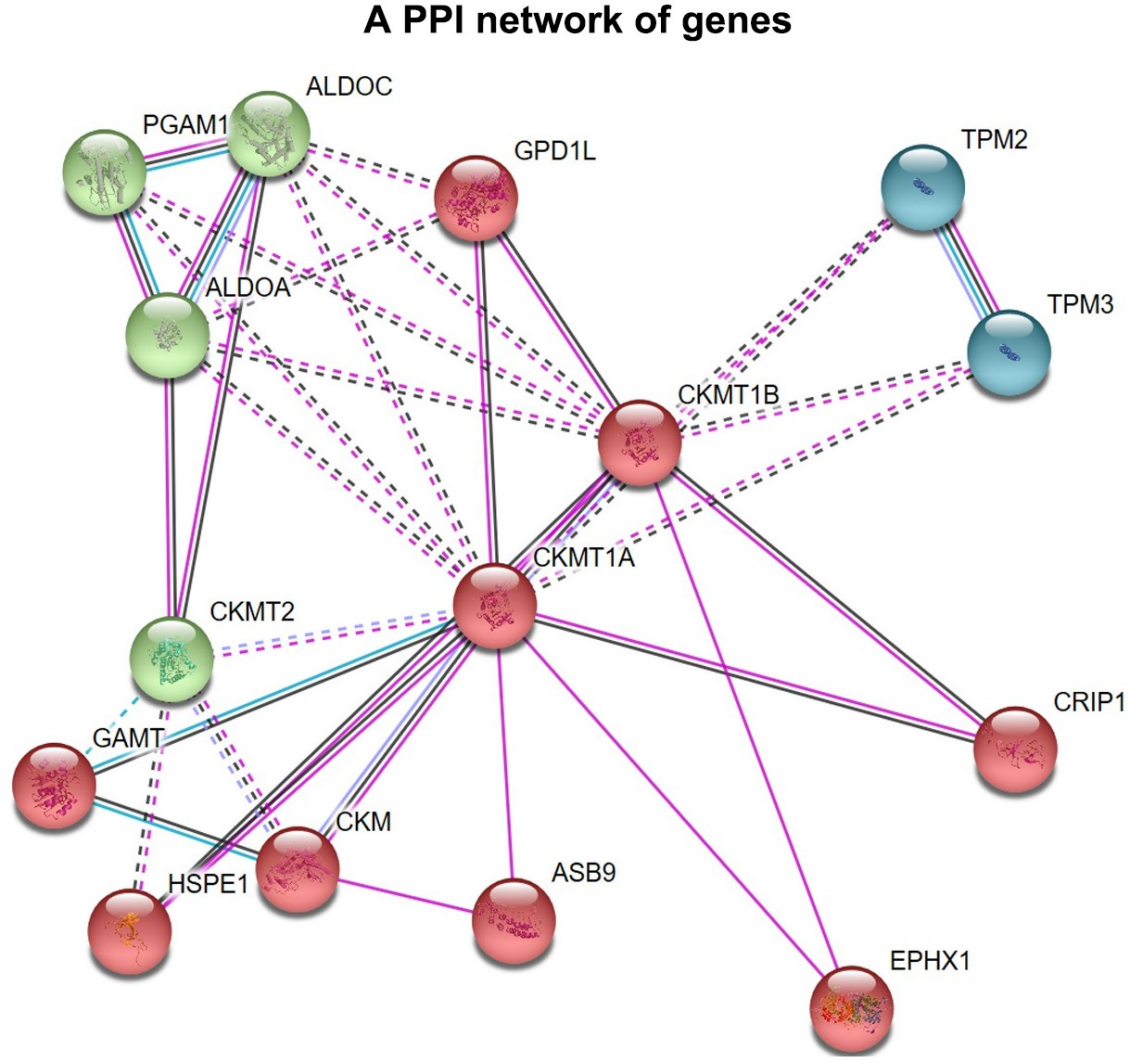
A protein-protein interaction (PPI) network shows the interaction between the screened DEGs. Each node represents one gene; the edge indicates the interaction relationship.

To further define the interactions between *CKMT1A* and other hub proteins, we constructed a PPI network of *CKMT1A* based on the information in the STRING protein query from public databases [21]. The PPI network consisted of 15 interacting nodes with 36 edges. It was concluded that creatine kinase, mitochondrial 1B(*CKMT1B*), glycerol-3-phosphate dehydrogenase 1 like (*GPD1L*), ankyrin repeat and SOCS box containing 9(*ASB9*), cysteine rich protein 1(*CRIP1*), creatine kinase (*CKM*), epoxide hydrolase 1(*EPHX1*), and guanidinoacetate N-methyltransferase (*GAMT*) were closely linked (Fig 3).

### Validated DEGs were associated with poor prognosis in TCGA

**Fig 4:**
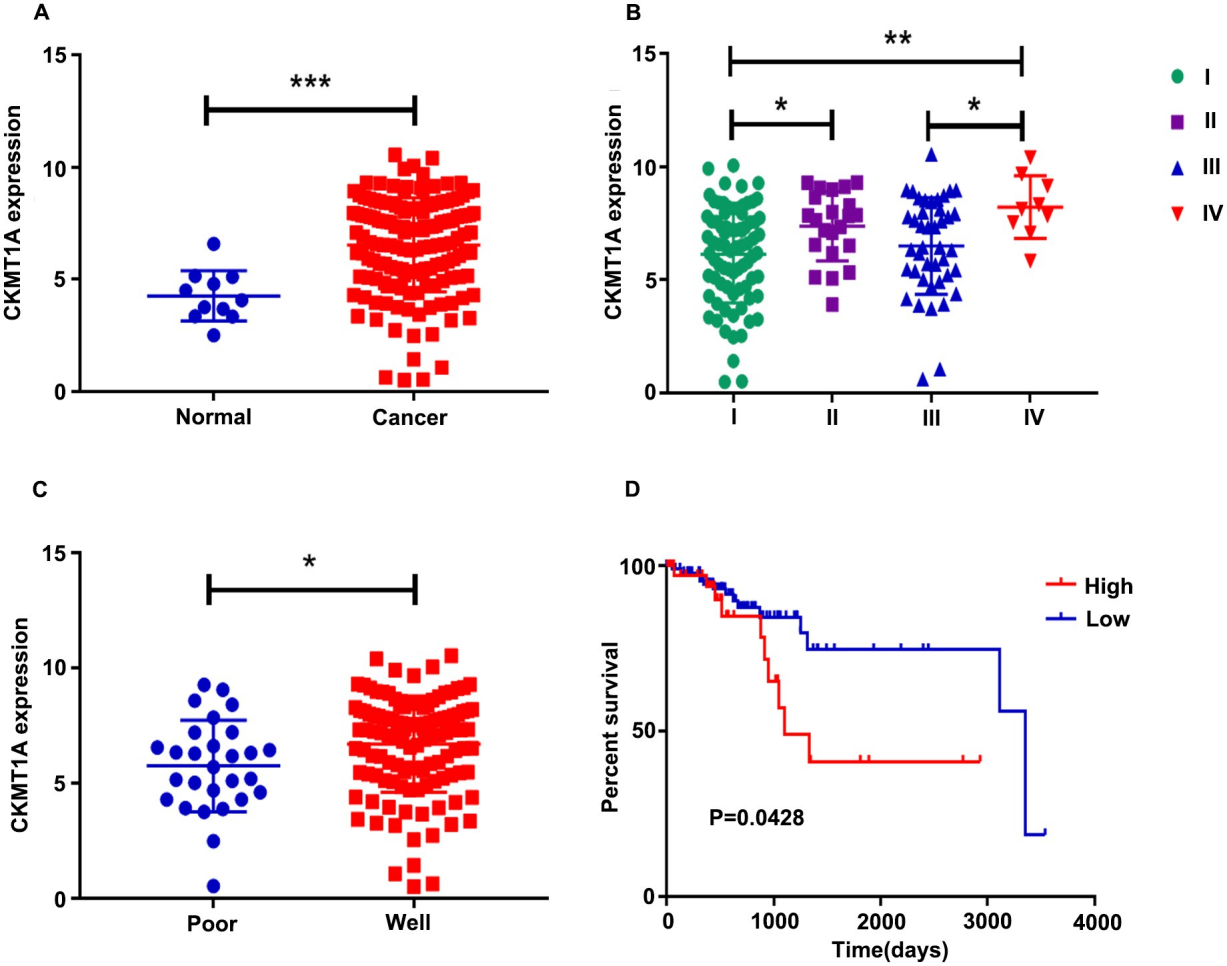
A higher expression of CKMT1A indicated poor prognosis in TCGA database. (A) mRNA expression of CKMT1A in cancer vs control from patients in TCGA database (***, P<0.001). (B) mRNA expression of CKMT1A in endometrial cancer patients at different FIGO stages of TCGA database (*, P<0.05; **, P<0.01). (C) mRNA expression of CKMT1A in endometrial cancer patients with histological differentiation of TCGA database (*, P<0.05). (D) Kaplan-Meier curve of CKMT1A was provided by patients from TCGA data.

To investigate the prognostic value of the identified DEG, *CKMT1A*, we further evaluated the association of *CKMT1A* expression levels with the overall survival of patients through Kaplan-Meier and log-rank analysis. Moreover, *CKMT1A* was upregulated in tumor tissues in TCGA (P = 0.005; Fig 4A) and differentially expressed at different FIGO stages (P = 0.005; Fig 4B). Meanwhile, the expression of *CKMT1A* was associated with a difference in histological grade (P = 0.030; Fig 4C). We divide them into High and Low groups according to the median survival time. A Kaplan-Meier survival analysis demonstrated that the higher expression of *CKMT1A*, the shorter the survival time (P = 0.043; Fig 4D). Therefore, a further research into *CKMT1A* was conducted, which showed its involvement in tumor processes of endometrial cancer and contributed to a more unbeneficial prognosis.

### GSEA analysis of *CKMT1A*

**Fig 5:**
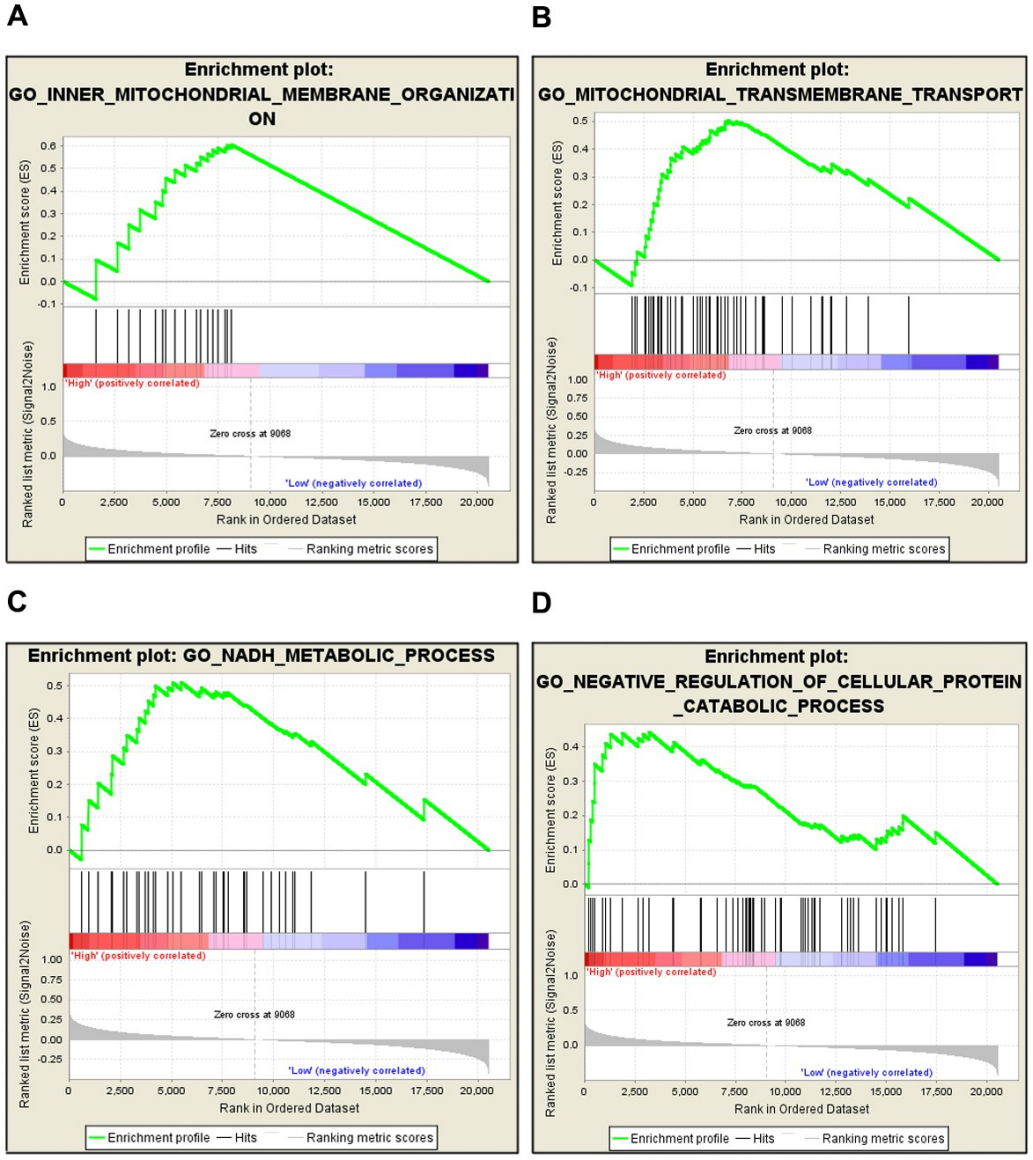
Gene set enrichment analysis (GSEA) analysis of CKMT1A. GSEA showed that CKMT1A was associated with (A) inner mitochondrial transmembrane organization; (B) mitochondrial transmembrane transport; (C) NADH metabolic process; and (D) negative regulation of cellular protein catabolic process.

According to the above results, we found that *CKMT1A* plays an important role at different stages of endometrial cancer. Therefore, it was necessary to elucidate the biological functions of *CKMT1A*. We performed a GSEA analysis using the GSEA software, a powerful tool used to deduce biological functions. The results showed that *CKMT1A* is associated with mitochondrial transmembrane transport, *NADH* metabolic processes, inner mitochondrial transmembrane organization, and negative regulation of cellular protein catabolic processes (Fig 5). These observations suggested that the expression level of *CKMT1A* may serve as a crucial regulator in the progression of endometrial cancer as well as a novel therapeutic option for patients with endometrial cancer so as to improve prognosis and prevent tumor progression.

### Analysis of *CKMT1A* expression in the Human Protein Atlas

**Fig 6:**
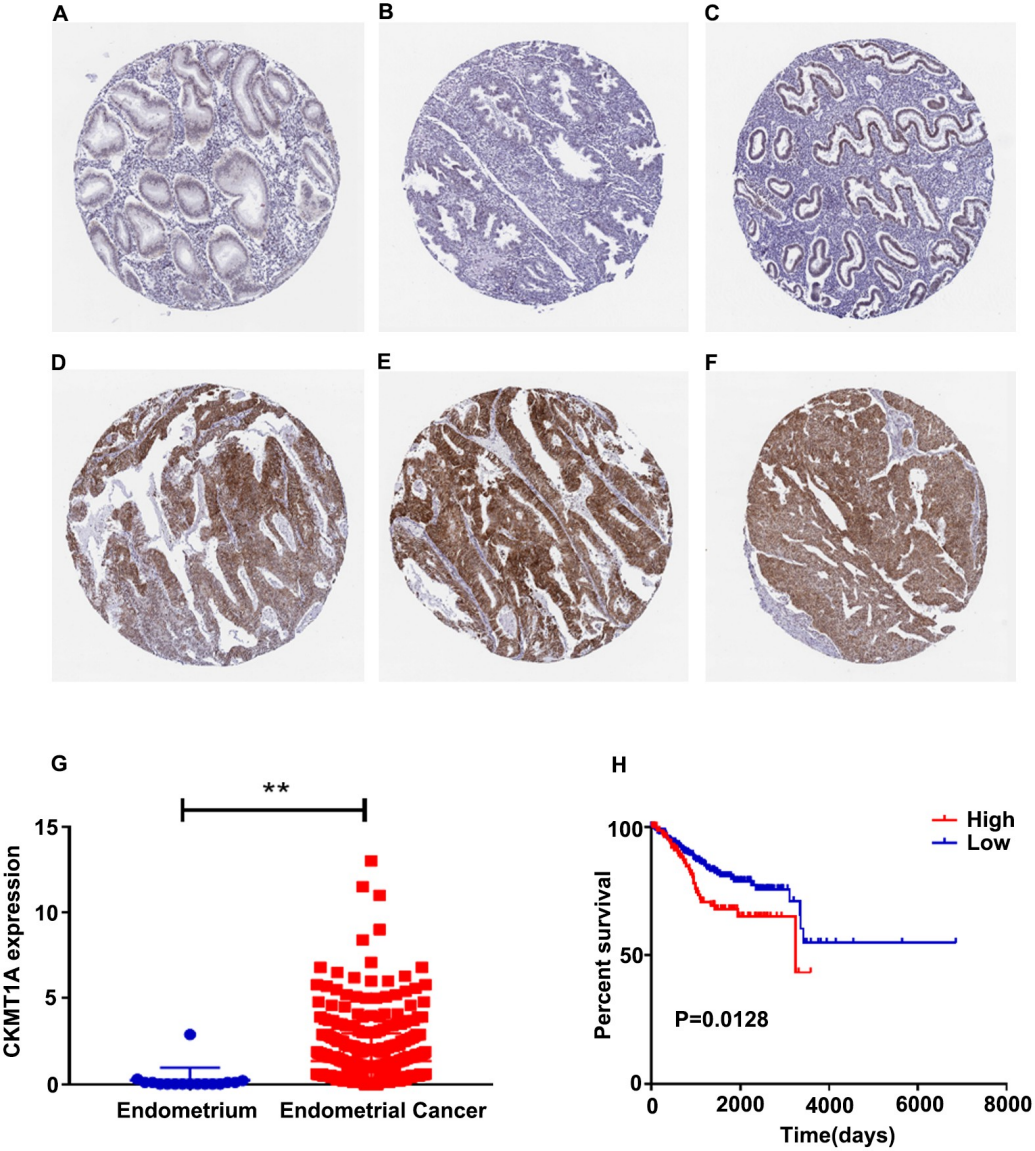
Analysis of CKMT1A in the Human Protein Atlas. (A)(B)(C) Expression of CKMT1A in normal tissues in the Human Protein Atlas. (D)(E)(F) Expression of CKMT1A in endometrial cancer samples in the Human Protein Atlas. (G) The expression of CKMT1A in endometrial cancer vs control from patients in the Human Protein Atlas (**, P<0.01). (H) Kaplan-Meier curve of CKMT1A was provided by patients from the Human Protein Atlas data.

To investigate the clinical significance of *CKMT1A* expression in patients with endometrial cancer, we used the Human Protein Atlas database to confirm the histological level of *CKMT1A*. The results suggested that *CKMT1A* is up-regulated in endometrial cancer tissue and down-regulated in the endometrium (P = 0.007; Fig 6A–6G). As expected, *CKMT1A* expression levels related to the prognosis of endometrial cancer as well as a higher level of *CKMT1A* demonstrated poorer prognosis than in patients with lower levels of expression (P = 0.0128; Fig 6H). Detailed sample material may be found in S6 and S7 Tables.

### Validation of clinical patients

**Fig 7:**
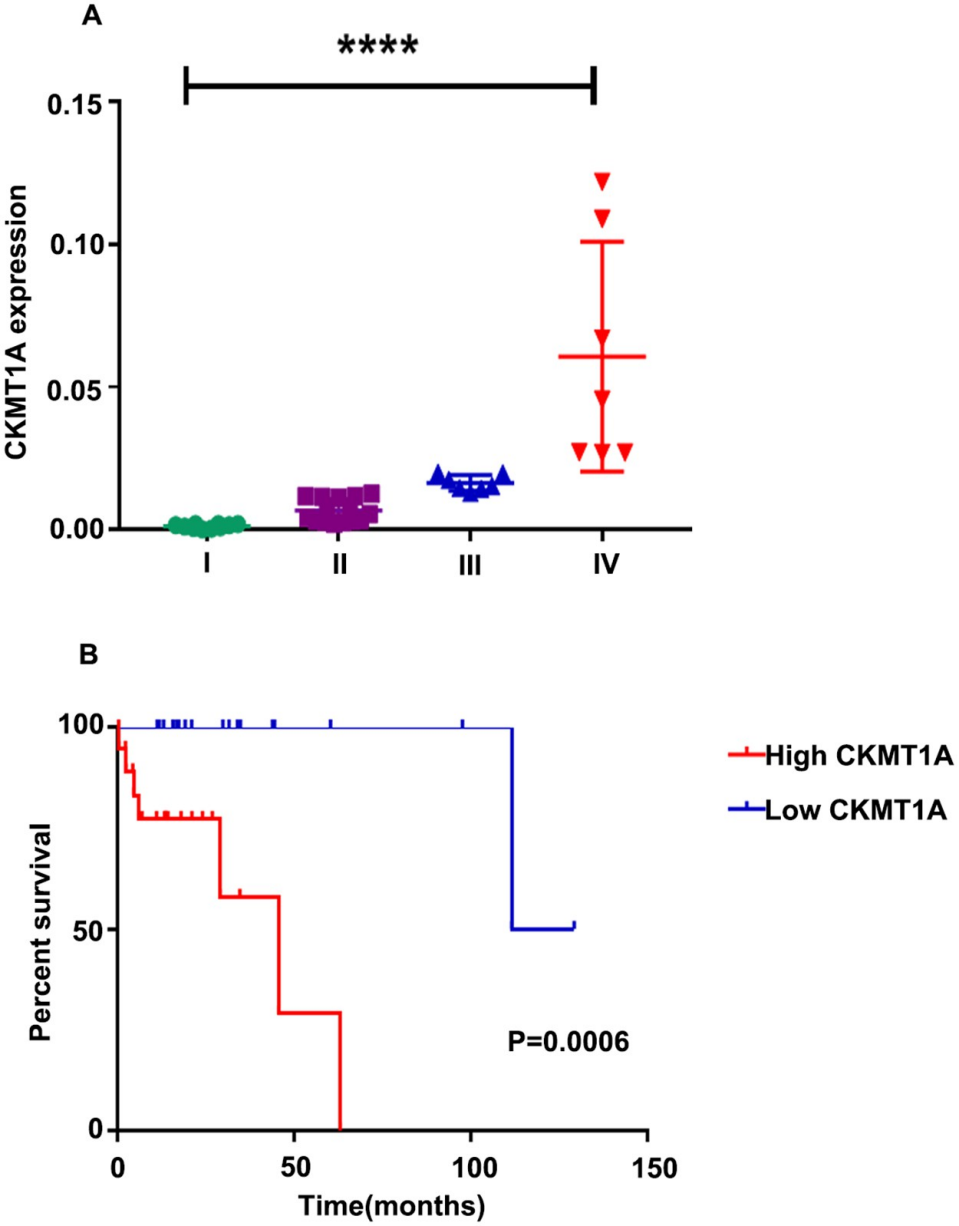
The expression of CKMT1A in association with the clinical characteristics and overall survival (OS) of patients with endometrial cancer. (A) The mRNA expression levels of CKMT1A in different groups of FIGO stages are displayed (****, P<0.0001). (B) Impact of CKMT1A expression on overall survival in clinical patients (n = 39).

**Fig 8:**
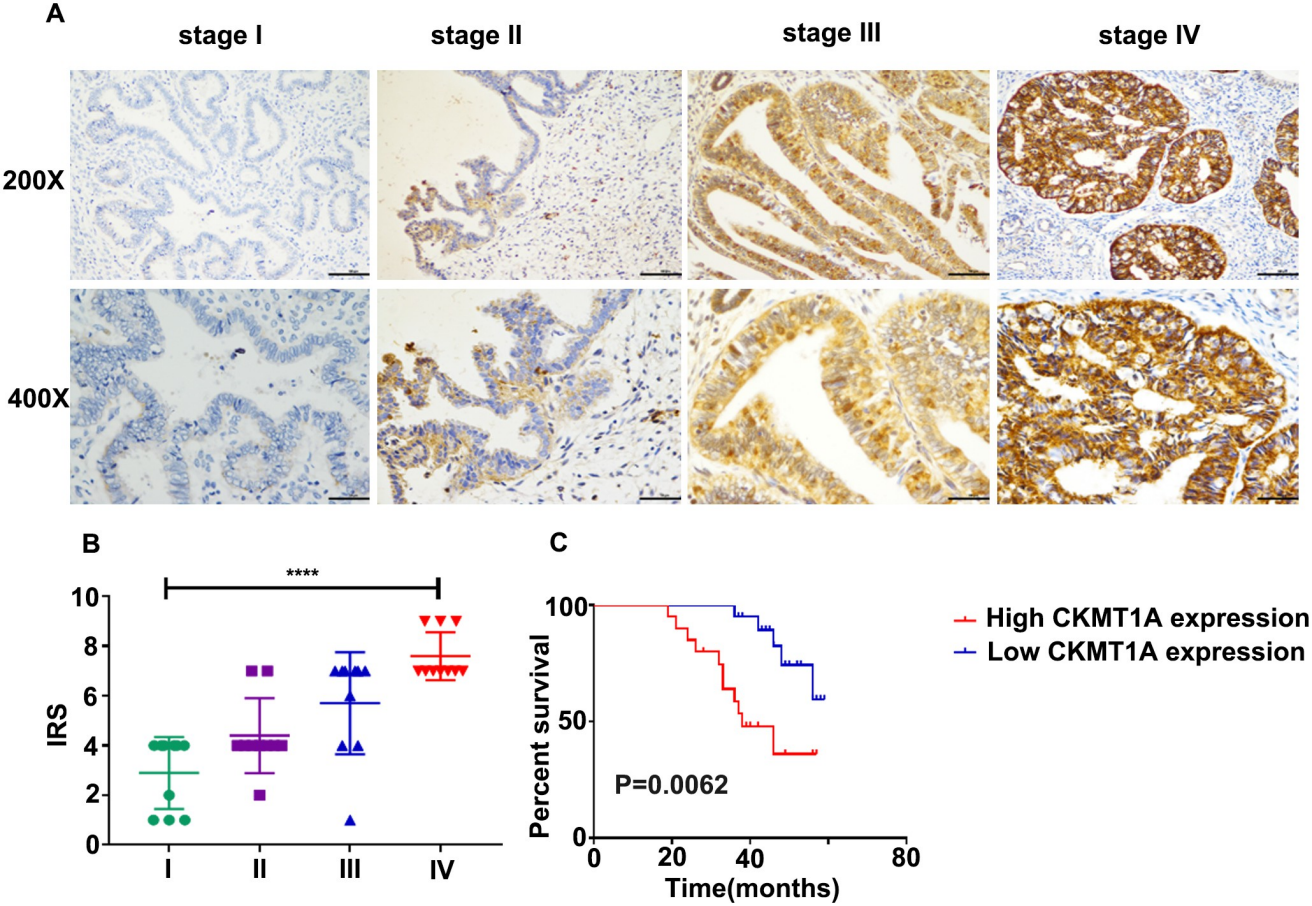
Immunohistochemical staining results of tumor and paratumor tissues in endometrial cancer with different stage. (A) Immunohistochemical staining results of tumor tissue in endometrial cancer with different stages (200× and 400×); (B) Immune responsive score (IRS) of CKMT1A in endometrial cancer different stage (n = 40); (C) Kaplan-Meier curves show the association between expression of CKMT1A and OS according to the immunohistochemical results (n = 40).

In order to further explore the clinical significance of the expression of *CKMT1A* in patients with endometrial cancer, the association between the expression of *CKMT1A* and various clinical features was investigated using real-time quantitative PCR in 39 endometrial cancer patients. The detailed clinicopathological data of the patients is shown in Table 1. *CKMT1A* expression was high in endometrial cancer patients at advanced stages, low histological differentiation and poor prognosis (P<0.0001; P = 0.006; Fig 7A and 7B). At the protein level, the results also showed that CKMT1A expression was higher in patients with advanced stage (Fig 8A and 8B). Next, 39 endometrial cancer samples were divided into “high” and “low” according to the *CKMT1A* median level of 0.008315392. We discovered that *CKMT1A* was closely correlated with the FIGO stage (P = 0.009), histological differentiation (P = 0.006) and abnormal CA125 value (P = 0.017) (Table 2). Therefore, *CKMT1A* was regarded as a prognostic marker associated with tumor progression in EC patients.

**Table 1:** Characteristics of patients with endometrial cancer.

Parameters	No. of cases	%
Age (years)		
< Median (56)	19	48.7
≥ Median (56)	20	51.3
BMI (kg/m^2^)		
< 24	19	48.7
≥ 24	20	51.3
Tumor size		
Small (<30 mm)	21	53.8
Large (≥30 mm)	18	46.2
Lymph node metastasis		
No	33	84.6
Yes	6	15.4
FIGO Stage		
I	10	25.6
II	15	38.6
III	7	17.9
IV	7	17.9
Histological differentiation		
Poor	17	43.6
Well	22	56.4
Myometrial invasion		
Surface	28	71.8
Deep	11	28.2
Menopause		
No	11	28.2
Yes	28	71.8

FIGO, International Federation of Gynecology and Obstetrics

**Table 2:** Association between CKMT1A expression and clinicopathological features of patients with endometrial cancer.

Parameters	Total	CKMT1A expression		X2	P Value
		High	Low		
Age				1.249	0.264
< 56	19	8	11		
≥ 56	20	12	8		
BMI (kg/m^2^)				2.092	0.148
< 24	19	12	7		
≥ 24	20	8	12		
Tumor size				1.293	0.256
<30 mm	21	9	12		
≥30 mm	18	11	7		
Lymph node metastasis				0.672	0.661^b^
N0	33	16	17		
N1	6	4	2		
FIGO Stage				8.816	0.009** b
I/II	25	6	19		
III/IV	14	14	0		
Histological differentiation				7.653	0.006**
Poor	17	13	4		
Well	22	7	15		
Myometrial invasion				0.936	0.333
Surface	28	13	15		
Deep	11	7	4		
Menopause				1.347	0.283^b^
No	11	6	5		
Yes	28	14	14		
CA 125				5.688	0.017*
Normal	28	13	15		
High	11	7	3		
CA 199				0.086	1.000^b^
Normal	30	15	15		
High	9	5	4		
AFP				0.312	0.703^b^
Normal	33	17	16		
High	6	3	3		
HE4				0.380	0.650
Normal	31	14	17		
High	8	6	2		

FIGO: International Federation of Gynecology and Obstetrics; CEA, carcinoembryonic antigen; CA, carbohydrate antigen;

^b^ indicates the Fisher test and the other test was the Chi-square test

Logistic regression analysis was conducted to examine the potential of *CKMT1A* expression levels in the prognosis of EC. As revealed by univariate analysis, tumor progression was associated with both high *CKMT1A* level [odds ratio; (OR = 3.459, P = 0.03)] and poor histological differentiation (OR = 0.144, P = 0.008). In a multivariate analysis, histological differentiation (OR = 6.90, P = 0.001) and FIGO stage (OR = 4.84, P = 0.02) were found to be the independent predictors of tumor progression among the patients with endometrial cancer (Table 3). More importantly, a multivariate Cox’s regression analysis suggested that *CKMT1A* level (HR = 0.388; P = 0.0001) and the difference of stage (HR = 1.174; P = 0.045) were independent prognostic factors for the OS of patients with endometrial cancer (Table 4).

**Table 3:** Logistic regression model analysis of stage predictors in patients with endometrial cancer.

Characteristics	Univariate			Multivariate		
	OR	95% CI	p Value	OR	95% CI	p Value
Age (≥56 vs<56)	2.062	0.575–7.393	0.266	1.496	0.512–4.372	0.462
Tumor size (≥30 mm vs <30 mm)	2.095	0.581–7.555	0.258	3.363	0.681–16.622	0.137
Lymph node metastasis (yes vs no)	2.125	0.341–13.241	0.419	1.521	0.832–6.712	0.414
Histological differentiation (well vs poor)	0.144	0.034–0.603	0.008**	6.900	2.140–22.240	0.001***
Myometrial invasion (deep vs surface)	2.019	0.481–8.485	0.337	11.382	0.937–138.297	0.056
Menopause (yes vs no)	0.833	0.206–3.376	0.798	5.041	1.281–19.841	0.121
CA125(>= 35 vs <35)	2.019	0.481–8.485	0.337	0.667	0.214–2.076	0.485
CA199(>= 37 vs <37)	1.250	0.280–5.585	0.770	0.217	0.047–1.007	0.051
AFP (>= 6.723 vs <6.723)	0.946	0.165–5.361	0.941	20.553	0.851–496.357	0.063
HE4(>= 140 vs <140)	3.643	0.633–20.956	0.148	0.201	0.032–1.276	0.089
CKMT1A (high vs low)	3.459	1.237–5.672	0.030*	4.84	1.27–18.42	0.020*

CEA, carcinoembryonic antigen; CA, carbohydrate antigen; OR, odds ratio; 95% CI, 95% confidence interval.

**Table 4:** Cox’s proportional hazard model analysis of prognostic factors in patients with endometrial cancer.

Characteristics	Univariate			Multivariate		
	HR	95% CI	p Value	HR	95% CI	p Value
Age (≥56 vs<56)	1.086	0.543–5.434	0.516	1.564	0.654–2.473	0.342
Tumor size (≥30 mm vs <30 mm)	2.135	0.731–4.525	0.348	2.454	0.782–4.125	0.534
Lymph node metastasis (yes vs no)	2.531	0.457–5.641	0.759	1.457	0.752–3.754	0.654
Stage number (III/IV vs. I/II)	3.354	1.012–5.354	0.036*	1.90	1.14–2.74	0.010*
Histological differentiation (well vs poor)	1.024	1.035–3.245	0.025*	1.98	1.45–2.43	0.041*
Myometrial invasion (deep vs surface)	3.512	0.497–3.525	0.677	1.364	0.563–2.235	0.052
Menopause (yes vs no)	0.635	0.713–5.468	0.867	5.041	1.281–6.854	0.354
CA125(>= 35 vs <35)	2.186	0.865–5.612	0.547	0.782	0.345–1.176	0.564
CA199(>= 37 vs <37)	1.560	0.780–6.465	0.870	1.674	0.453–1.127	0.061
AFP (>= 6.723 vs <6.723)	0.687	0.135–1.536	0.941	2.568	0.454–4.754	0.086
HE4(>= 140 vs <140)	2.678	0.687–6.357	0.068	0.201	0.347–1.754	0.078
CKMT1A (high vs low)	3.755	1.455–5.867	0.042*	2.35	1.280–3.421	0.031*

Moreover, both Kaplan-Meier survival analysis and log-rank analysis were carried out to demonstrate that the higher levels of *CKMT1A* expression were closely associated with the lower chance of survival for the patients with endometrial cancer (Figs 7C and 8C). In immunohistochemistry, there were 40 endometrial cancer samples classed into “high” or “low” according to the median immunohistochemistry score. Essentially, the obtained results imply that *CKMT1A* expression plays a significant role in predicting tumor progression in patients with endometrial cancer.

## Discussion

In relation to EC patients at advanced stages of disease, treatment options are limited and the prognosis is unsatisfactory [23]. Thus, it is essential to identify an effective indicator to predict the prognosis of endometrial cancer in order to provide novel therapeutic methods. High-throughput technologies have been increasingly used as an important tool in life science, such as cancer staging, early cancer diagnosis, and the assessment of prognosis [24]. Many promising novel biomarkers and therapeutic targets have been explored by analyzing the transcriptomes of various tumors. However, the genetic expression characteristics related to the EC stage have not been fully explored. Therefore, the identification of novel biomarkers related to the EC stage is of great significance to predicting the prognosis of patients at different stages of EC with the hopes of extending patient survival. In our study, we obtained genetic expression data of EC from TCGA database and identified a novel gene related to the prognosis of endometrial cancer.

In order to identify new biomarkers for the prognosis of endometrial cancer, we compared the mRNA expression in stage II vs. stage I and stage IV vs. stage III in the TCGA database. Thereafter, the association between mRNA expression and prognosis was investigated, for which *CKMT1A* was identified as a potential biomarker that was closely related to poor prognosis. GO and GSEA were used to further assess the functions in the signaling pathways of *CKMT1A* in terms of promoting disease progression, and the role of *CKMT1A* within the tumor microenvironment was highlighted. We verified that the gene was closely associated with metabolic processes of the following compounds: phosphate-containing compounds, small molecules, carbohydrate derivates, organophosphates, carboxylic acids, nucleobases containing small molecules, purine-containing compounds, carbohydrates, carbon, biosynthesis of amino acids, glycolysis/gluconeogenesis, arginine and proline, glycine, serine and threonine, fructose and mannose, and the pentose phosphate pathway. One mechanism in endometrial cancer development was found to be the down-regulation in the uptake of amino acids, glucose, *NADPH* production. Moreover, the utilization of intermediates from glycolysis as well as the tricarboxylic acid cycle have been considered major contributors to tumorigenesis [25, 26]. In our study, a pathway analysis identified that the biosynthesis of amino acids, metabolism of glycine, serine and threonine, glycolysis/gluconeogenesis and the pentose phosphate signaling pathway were important in EC progression. This result is supported by the idea that metabolomics is widely used in cancer metabolism and biomarker identification to infer the progression of cancer [27–29]. In addition, estradiol metabolites may serve as biomarkers in endometrial cancer prognosis [30]. Finally, the clinical specimens of the EC patients were analyzed to confirm that *CKMT1A* expression was associated with poor survival. Accordingly, *CKMT1A* was found to be upregulated in EC patients with advanced stages of cancer. EC patients with high *CKMT1A* expression had poor survival.

*CKMT1A* can reduce the arrest of the G2/M phase cell cycle, enhance colony formation rates, lower the apoptosis rate and *c-PARP* level, and elevate *STAT3* phosphorylation levels via *CRISPR/Cas9* system in nasopharyngeal carcinoma [19]. Previously, a study demonstrated that the phosphocreatine energy shuttle creates a druggable metabolic vulnerability via *MtCK1Y153* phosphorylation in *HER2* breast cancer [16]. Moreover, the n335586/miR-924/*CKMT1A* axis was found to contribute to HCC cell migration and invasion [17]. Furthermore, a promising therapeutic modality in the EVI1-driven AML subtype was observed to target *CKMT1* [15]. Recent studies have reported that *CKMT1* and *NCOA1* are biomarkers for survival in head and neck squamous cell carcinoma [18]. However, no previous study focused on the relationship between the expression of *CKMT1A* in EC and the prognosis of patients with EC.

In this study, we found that the expression of *CKMT1A* was closely related to better survival from the TCGA database. Furthermore, we verified the clinical significance of *CKMT1A* expression levels in Human Protein Atlas and clinical endometrial cancer samples. A higher expression of *CKMT1A* was consistently found to be associated with the FIGO stage, histological differentiation and abnormal *CA125* value, which signified a poor prognosis for patients with endometrial cancer. Aligning with similar results showing that *CKMT1A* lowers the apoptosis rate and promotes proliferation and migration in nasopharyngeal carcinoma and neck squamous cell carcinoma [18, 19], so that we deduced that *CKMT1A* plays an essential role in regulating endometrial cancer progression.

To the best of our knowledge, this study is the first to identify *CKMT1A* as a novel prognostic biomarker in EC patients at different stages of disease through the analysis of bioinformatics and confirmation within the Human Protein Atlas and clinical samples. Moreover, *CKMT1A* expression levels increase with advanced stages. A higher expression of *CKMT1A* contributed to tumor progression, which may contribute to unfavorable clinical outcomes. The expression of *CKMT1A* may be a valuable adjuvant parameter in the prognosis of patients with endometrial cancer and provides a potential future therapeutic strategy.

## Supporting information

S1 Table Sample materials in TCGA. (DOC)

S2 Table Expression of 63 genes specific for FIGO stages II and I in TCGA. LogFC: FoldChange.
(DOC)

S3 Table Table S3 expression of 171 genes specific for FIGO stages IV and III in TCGA. LogFC: FoldChange.
(DOC)

S4 Table Results of gene set enrichment analysis with differentially expressed genes between CKMT1A-high and CKMT1A-low. BP: Biological Process; MF: Molecular Function; CC: Cellular Component; FDR: false discovery rate.
(DOC)

S5 Table Results of pathway annotation with differentially expressed genes between CKMT1A-high and CKMT1A-low. FDR: false discovery rate.
(DOC)

S6 Table Endometrium sample materials in the Human Protein Atlas. (DOC)

S7 Table The samples material of endometrial cancer in the Human Protein Atlas. (DOC)
