# Supplementary material for: CKMT1A is a novel potential prognostic biomarker in patients with endometrial cancer
Source: PLoS One. 2022 Jan 25;17(1):e0262000. doi: 10.1371/journal.pone.0262000 (PMC8789190; doi:10.1371/journal.pone.0262000)
Supplement: S1 Table — (DOC) [file pone.0262000.s001.doc]

**S1 Table.** Sample materials in TCGA

| Samples | FIGO stages | Samples | FIGO stages |
| --- | --- | --- | --- |
| TCGA-PG-A916-01 | Stage I | TCGA-AJ-A3BK-01 | Stage I |
| TCGA-A5-A2K2-01 | Stage I | TCGA-AX-A2IN-01 | Stage I |
| TCGA-EO-A3KX-01 | Stage I | TCGA-EY-A1GL-01 | Stage II |
| TCGA-AX-A3G6-01 | Stage I | TCGA-EO-A3KU-01 | Stage II |
| TCGA-KP-A3W1-01 | Stage I | TCGA-B5-A0K9-01 | Stage II |
| TCGA-B5-A1MW-01 | Stage I | TCGA-AJ-A2QM-01 | Stage II |
| TCGA-D1-A3JP-01 | Stage I | TCGA-B5-A3FC-01 | Stage II |
| TCGA-AJ-A3EL-01 | Stage I | TCGA-AJ-A3I9-01 | Stage II |
| TCGA-AJ-A3EK-01 | Stage I | TCGA-A5-A2K7-01 | Stage II |
| TCGA-AJ-A3BG-01 | Stage I | TCGA-QF-A5YS-01 | Stage II |
| TCGA-AX-A3G8-01 | Stage I | TCGA-B5-A11R-01 | Stage II |
| TCGA-EY-A2OP-01 | Stage I | TCGA-BK-A6W3-01 | Stage II |
| TCGA-EO-A3KW-01 | Stage I | TCGA-PG-A5BC-01 | Stage II |
| TCGA-EO-A3AS-01 | Stage I | TCGA-EO-A3AU-01 | Stage II |
| TCGA-D1-A3DH-01 | Stage I | TCGA-AJ-A3NG-01 | Stage II |
| TCGA-AX-A2HH-01 | Stage I | TCGA-EY-A72D-01 | Stage II |
| TCGA-EY-A549-01 | Stage I | TCGA-AJ-A3OJ-01 | Stage II |
| TCGA-EY-A548-01 | Stage I | TCGA-QF-A5YT-01 | Stage II |
| TCGA-EY-A1GP-01 | Stage I | TCGA-AJ-A6NU-01 | Stage II |
| TCGA-AJ-A3OK-01 | Stage I | TCGA-BG-A3PP-01 | Stage II |
| TCGA-AJ-A3NE-01 | Stage I | TCGA-DI-A1BU-01 | Stage II |
| TCGA-A5-A2K3-01 | Stage I | TCGA-DI-A2QY-01 | Stage II |
| TCGA-PG-A7D5-01 | Stage I | TCGA-AX-A3FX-01 | Stage II |
| TCGA-EY-A2OQ-01 | Stage I | TCGA-BG-A0MK-01 | Stage II |
| TCGA-B5-A3FH-01 | Stage I | TCGA-2E-A9G8-01 | Stage III |
| TCGA-FI-A3PV-01 | Stage I | TCGA-BG-A3EW-01 | Stage III |
| TCGA-KJ-A3U4-01 | Stage I | TCGA-A5-A2K4-01 | Stage III |
| TCGA-EY-A3L3-01 | Stage I | TCGA-AX-A3G3-01 | Stage III |
| TCGA-B5-A3FD-01 | Stage I | TCGA-EO-A22X-01 | Stage III |
| TCGA-AJ-A3NC-01 | Stage I | TCGA-A5-A1OH-01 | Stage III |
| TCGA-AJ-A5DW-01 | Stage I | TCGA-AJ-A3OL-01 | Stage III |
| TCGA-EY-A547-01 | Stage I | TCGA-AJ-A3QS-01 | Stage III |
| TCGA-5B-A90C-01 | Stage I | TCGA-QS-A8F1-01 | Stage III |
| TCGA-AJ-A3BI-01 | Stage I | TCGA-KP-A3W4-01 | Stage III |
| TCGA-E6-A2P9-01 | Stage I | TCGA-EY-A1GX-01 | Stage III |
| TCGA-AJ-A8CT-01 | Stage I | TCGA-FI-A2EY-01 | Stage III |
| TCGA-BK-A6W4-01 | Stage I | TCGA-QS-A5YQ-01 | Stage III |
| TCGA-EO-A3L0-01 | Stage I | TCGA-5S-A9Q8-01 | Stage III |
| TCGA-AJ-A8CW-01 | Stage I | TCGA-AX-A2H4-01 | Stage III |
| TCGA-A5-A2K5-01 | Stage I | TCGA-AX-A3FV-01 | Stage III |
| TCGA-AX-A3FS-01 | Stage I | TCGA-AJ-A23N-01 | Stage III |
| TCGA-BK-A4ZD-01 | Stage I | TCGA-QS-A744-01 | Stage III |
| TCGA-AJ-A5DV-01 | Stage I | TCGA-QS-A5YR-01 | Stage III |
| TCGA-PG-A915-01 | Stage I | TCGA-AX-A3FZ-01 | Stage III |
| TCGA-EO-A22Y-01 | Stage I | TCGA-B5-A5OC-01 | Stage III |
| TCGA-PG-A914-01 | Stage I | TCGA-AX-A3G9-01 | Stage III |
| TCGA-PG-A6IB-01 | Stage I | TCGA-DI-A2QT-01 | Stage III |
| TCGA-PG-A917-01 | Stage I | TCGA-AX-A3GI-01 | Stage III |
| TCGA-A5-A3LP-01 | Stage I | TCGA-AX-A3G4-01 | Stage III |
| TCGA-AJ-A3TW-01 | Stage I | TCGA-AJ-A3NF-01 | Stage III |
| TCGA-AJ-A3IA-01 | Stage I | TCGA-B5-A5OD-01 | Stage III |
| TCGA-AJ-A2QO-01 | Stage I | TCGA-EO-A3AV-01 | Stage III |
| TCGA-AJ-A3NH-01 | Stage I | TCGA-D1-A3DA-01 | Stage III |
| TCGA-BK-A139-02 | Stage I | TCGA-B5-A3S1-01 | Stage III |
| TCGA-D1-A2G0-01 | Stage I | TCGA-AP-A5FX-01 | Stage III |
| TCGA-EY-A2OO-01 | Stage I | TCGA-EY-A1GO-01 | Stage III |
| TCGA-AX-A1C7-01 | Stage I | TCGA-EY-A4KR-01 | Stage III |
| TCGA-EO-A22U-01 | Stage I | TCGA-JU-AAVI-01 | Stage III |
| TCGA-EO-A3AY-01 | Stage I | TCGA-EO-A3B0-01 | Stage III |
| TCGA-AJ-A3BH-01 | Stage I | TCGA-AJ-A3EJ-01 | Stage III |
| TCGA-E6-A8L9-01 | Stage I | TCGA-EY-A210-01 | Stage III |
| TCGA-AJ-A3EM-01 | Stage I | TCGA-EY-A54A-01 | Stage III |
| TCGA-AX-A3FW-01 | Stage I | TCGA-FI-A3PX-01 | Stage III |
| TCGA-BK-A56F-01 | Stage I | TCGA-AJ-A3BD-01 | Stage III |
| TCGA-AX-A3FT-01 | Stage I | TCGA-AJ-A3BF-01 | Stage III |
| TCGA-EY-A5W2-01 | Stage I | TCGA-B5-A5OE-01 | Stage III |
| TCGA-EO-A3B1-01 | Stage I | TCGA-KP-A3VZ-01 | Stage IV |
| TCGA-AX-A3GB-01 | Stage I | TCGA-E6-A2P8-01 | Stage IV |
| TCGA-EO-A1Y7-01 | Stage I | TCGA-DF-A2KY-01 | Stage IV |
| TCGA-BS-A0V4-01 | Stage I | TCGA-EY-A3QX-01 | Stage IV |
| TCGA-BS-A0V7-01 | Stage I | TCGA-AP-A3K1-01 | Stage IV |
| TCGA-KP-A3W0-01 | Stage I | TCGA-B5-A0JN-01 | Stage IV |
| TCGA-A5-A3LO-01 | Stage I | TCGA-D1-A3DG-01 | Stage IV |
| TCGA-B5-A3FB-01 | Stage I | TCGA-AX-A3G7-01 | Stage IV |
| TCGA-EO-A3AZ-01 | Stage I | TCGA-K6-A3WQ-01 | Stage IV |
| TCGA-BK-A13B-01 | Stage I | TCGA-EY-A2ON-01 | Stage IV |
| TCGA-SL-A6JA-01 | Stage I | TCGA-FL-A3WE-11 | adjacent normal tissues |
| TCGA-SJ-A6ZJ-01 | Stage I | TCGA-FL-A1YN-11 | adjacent normal tissues |
| TCGA-KP-A3W3-01 | Stage I | TCGA-FL-A1YF-11 | adjacent normal tissues |
| TCGA-DI-A1C3-01 | Stage I | TCGA-FL-A1YQ-11 | adjacent normal tissues |
| TCGA-B5-A3F9-01 | Stage I | TCGA-FL-A1YU-11 | adjacent normal tissues |
| TCGA-SJ-A6ZI-01 | Stage I | TCGA-FL-A1YV-11 | adjacent normal tissues |
| TCGA-DF-A2KZ-01 | Stage I | TCGA-FL-A1YI-11 | adjacent normal tissues |
| TCGA-DF-A2KV-01 | Stage I | TCGA-FL-A1YH-11 | adjacent normal tissues |
| TCGA-H5-A2HR-01 | Stage I | TCGA-FL-A1YL-11 | adjacent normal tissues |
| TCGA-AX-A3G1-01 | Stage I | TCGA-FL-A1YT-11 | adjacent normal tissues |
| TCGA-B5-A1MS-01 | Stage I | TCGA-FL-A1YG-11 | adjacent normal tissues |
| TCGA-B5-A3FA-01 | Stage I |  |  |
