# Supplementary material for: CKMT1A is a novel potential prognostic biomarker in patients with endometrial cancer
Source: PLoS One. 2022 Jan 25;17(1):e0262000. doi: 10.1371/journal.pone.0262000 (PMC8789190; doi:10.1371/journal.pone.0262000)
Supplement: S2 Table — LogFC: FoldChange. (DOC) [file pone.0262000.s002.doc]

**S2 Table.** Expression of 63 genes specific for FIGO stages II and I in TCGA

| Gene symbol | logFC | p-value | Style |
| --- | --- | --- | --- |
| MYO3B | 1.04 | 0.049959 | up |
| NRXN1 | 1.12 | 0.02033 | up |
| HOXA13 | 1.32 | 0.001497 | up |
| C3orf57 | 2.04 | 0.000738 | up |
| AKR1B10 | 1.85 | 0.026174 | up |
| AKR1B15 | 1.26 | 0.031644 | up |
| DACT1 | 1.01 | 0.010352 | up |
| ACSS3 | 1.24 | 0.031887 | up |
| PCSK6 | 1.51 | 0.000563 | up |
| LASS3 | 1.22 | 0.005355 | up |
| C3orf14 | 1.4 | 0.003972 | up |
| SPAG17 | 1.42 | 0.031399 | up |
| CSMD2 | 1.04 | 0.016702 | up |
| LOC285629 | 1.25 | 0.013049 | up |
| KRT14 | 1.55 | 0.043959 | up |
| AOX1 | 1.05 | 0.015429 | up |
| EDIL3 | 1.1 | 0.032401 | up |
| MUM1L1 | 1.32 | 0.0059 | up |
| SPAG16 | 1.18 | 0.025826 | up |
| SLC1A2 | 1.24 | 0.004039 | up |
| CKMT1A | 1.12 | 0.027266 | up |
| ATP10B | 1.45 | 0.01765 | up |
| SP9 | 1.23 | 0.037295 | up |
| CHRM2 | 1.07 | 0.001023 | up |
| SCNN1B | 1.41 | 0.048729 | up |
| L1TD1 | 1.04 | 0.0052 | up |
| SNCAIP | 1.08 | 0.003078 | up |
| SCN9A | 1.1 | 0.037617 | up |
| LOC554202 | 1.84 | 0.002332 | up |
| ZFHX4 | 1.22 | 0.042063 | up |
| COL8A1 | 1.13 | 0.021774 | up |
| CWH43 | 1.74 | 0.010786 | up |
| SFRP4 | 1.48 | 0.040608 | up |
| SFRP1 | 1.02 | 0.026916 | up |
| KLK12 | 1.38 | 0.043226 | up |
| LRRC55 | 1.11 | 0.0204 | up |
| IFNE | 1.16 | 0.013599 | up |
| GJB6 | 1.63 | 0.00959 | up |
| STYK1 | 1.13 | 0.012817 | up |
| PCDH10 | 1.44 | 0.018458 | up |
| FREM2 | 1.12 | 0.046386 | up |
| ATRNL1 | 1.25 | 0.029267 | up |
| OPRK1 | 1.48 | 0.017641 | up |
| PRDM6 | 1.13 | 0.013866 | up |
| VWA2 | 1.3 | 0.031726 | up |
| SERTAD4 | 1.05 | 0.041401 | up |
| DLX6AS | 1.23 | 0.043792 | up |
| NEFM | 1.03 | 0.004719 | up |
| PITX1 | 1.1 | 0.038853 | up |
| SLURP1 | 1.24 | 0.020608 | up |
| C14orf50 | 1.13 | 0.006783 | up |
| STMN2 | 1.21 | 0.031406 | up |
| ALX1 | 1.15 | 0.018442 | up |
| C12orf56 | 1.06 | 0.033725 | up |
| SLC17A7 | -1.26 | 0.021488 | down |
| CPNE7 | -1.07 | 0.021988 | down |
| CXCR2P1 | -1.06 | 0.032632 | down |
| HIST1H3G | -1.07 | 0.036826 | down |
| CTSW | -1.01 | 0.025007 | down |
| FOLR3 | -1.11 | 0.017915 | down |
| SNCG | -1.1 | 0.038349 | down |
| ALOX15B | -1.04 | 0.020319 | down |
| ANGPTL1 | -1.34 | 0.035517 | down |

LogFC: FoldChange
