# Supplementary material for: CKMT1A is a novel potential prognostic biomarker in patients with endometrial cancer
Source: PLoS One. 2022 Jan 25;17(1):e0262000. doi: 10.1371/journal.pone.0262000 (PMC8789190; doi:10.1371/journal.pone.0262000)
Supplement: S3 Table — LogFC: FoldChange. (DOC) [file pone.0262000.s003.doc]

**S3 Table.** Expression of 171 genes specific for FIGO stages IV and III in TCGA

| Gene symbol | logFC | p-value | Style |
| --- | --- | --- | --- |
| SGSM1 | 1.69 | 2.37E-02 | up |
| RND2 | 1.28 | 4.58E-02 | up |
| SYT4 | 1.43 | 4.64E-02 | up |
| NPPC | 1.23 | 3.60E-02 | up |
| TNNT1 | 2.32 | 4.10E-02 | up |
| KRT15 | 1.75 | 2.22E-02 | up |
| RPS6KA6 | 1.8 | 3.38E-02 | up |
| CKMT1A | 1.49 | 2.82E-02 | up |
| CKMT1B | 1.41 | 3.92E-02 | up |
| KCNK3 | 1.89 | 1.24E-02 | up |
| USP44 | 1.31 | 4.82E-02 | up |
| SYNGR3 | 1.11 | 3.80E-02 | up |
| C9orf66 | 1.52 | 7.03E-03 | up |
| TDRD10 | 1.38 | 2.94E-02 | up |
| MAGEA9B | 2.8 | 4.67E-02 | up |
| CHST13 | 1.67 | 4.60E-03 | up |
| DQX1 | 1.7 | 2.25E-03 | up |
| XCL1 | 1.45 | 4.22E-02 | up |
| PRL | 2.19 | 5.71E-04 | up |
| WIPF3 | 1.17 | 2.75E-02 | up |
| LOC100132111 | 1.07 | 1.64E-02 | up |
| PCDHA10 | 2.79 | 6.87E-03 | up |
| CHCHD10 | 1.17 | 5.55E-03 | up |
| DSG1 | 2.12 | 5.31E-03 | up |
| TMEM195 | 1.97 | 5.91E-05 | up |
| ENHO | 1.8 | 2.05E-02 | up |
| ZNF454 | 1.16 | 2.91E-02 | up |
| PCDH15 | 1.46 | 4.18E-02 | up |
| DSC1 | 1.6 | 1.40E-03 | up |
| NXF5 | 1.42 | 2.85E-02 | up |
| KCNK12 | 1.35 | 8.26E-03 | up |
| SULT1E1 | 1.79 | 3.14E-02 | up |
| ADSSL1 | 1.19 | 6.78E-03 | up |
| HCLS1 | 1.16 | 4.18E-02 | up |
| SOX30 | 1.06 | 4.93E-02 | up |
| GABBR2 | 1.58 | 2.85E-02 | up |
| HTR3A | 2.17 | 3.42E-02 | up |
| GDAP1L1 | 1.32 | 9.48E-03 | up |
| KIF1A | 2.53 | 1.23E-02 | up |
| FAM123C | 1.22 | 6.31E-03 | up |
| CALB2 | 2.04 | 4.59E-02 | up |
| MAGEA4 | 3.73 | 6.17E-03 | up |
| HOXD1 | 2.97 | 9.70E-03 | up |
| CTAG2 | 1.7 | 3.28E-02 | up |
| CEACAM5 | 2.3 | 4.31E-02 | up |
| FZD9 | 1.25 | 3.10E-02 | up |
| FAM27B | 1.58 | 1.85E-04 | up |
| DGKB | 1.65 | 1.34E-03 | up |
| SLC6A3 | 1.4 | 2.15E-02 | up |
| SFTPA1 | 1.2 | 1.19E-02 | up |
| FAM9C | 1.5 | 4.87E-02 | up |
| CBS | 1.56 | 3.39E-02 | up |
| NTS | 3.56 | 6.54E-03 | up |
| KCNU1 | 1.47 | 5.96E-03 | up |
| SOX2 | 2.2 | 3.36E-02 | up |
| PLA2G16 | 1.41 | 1.23E-02 | up |
| RHOXF2B | 1.83 | 1.03E-02 | up |
| CAND2 | 1.24 | 1.57E-02 | up |
| ITGA9 | -1.34 | 2.95E-02 | down |
| CCDC116 | -1.02 | 1.84E-02 | down |
| STXBP6 | -1.63 | 1.54E-02 | down |
| ITGB1BP2 | -1.04 | 1.48E-02 | down |
| GSDMA | -1.58 | 1.25E-02 | down |
| MYO1H | -1.05 | 2.55E-02 | down |
| COL14A1 | -1.52 | 3.69E-02 | down |
| LOC284276 | -1.32 | 4.89E-02 | down |
| AKAP3 | -1.16 | 3.67E-02 | down |
| CCL22 | -1.45 | 3.04E-02 | down |
| C8orf48 | -1.2 | 4.20E-02 | down |
| ZNF831 | -1.3 | 3.96E-02 | down |
| RAB33A | -1.09 | 2.56E-02 | down |
| MMP23B | -1.12 | 4.49E-02 | down |
| HFE | -1.05 | 4.65E-02 | down |
| ITGBL1 | -1.61 | 2.84E-02 | down |
| LOC100271722 | -1.17 | 7.76E-03 | down |
| SLC16A6 | -1.01 | 2.64E-02 | down |
| ELAVL2 | -1.45 | 1.76E-02 | down |
| MAEL | -1.02 | 1.43E-02 | down |
| DYNC1I1 | -2.24 | 8.08E-03 | down |
| CSMD1 | -1.74 | 4.60E-02 | down |
| C1orf130 | -1.41 | 2.52E-02 | down |
| GPR143 | -1.31 | 2.21E-02 | down |
| ODZ2 | -1.9 | 1.27E-02 | down |
| PCYT1B | -2.05 | 1.97E-02 | down |
| SIPA1L2 | -1.07 | 1.77E-02 | down |
| C2orf63 | -1.29 | 1.85E-04 | down |
| TCTEX1D2 | -1.07 | 1.24E-02 | down |
| RNF175 | -1.51 | 3.18E-02 | down |
| GPR55 | -1.33 | 2.30E-02 | down |
| DPY19L2P4 | -1.04 | 4.05E-02 | down |
| MYLK4 | -1 | 2.21E-02 | down |
| ARRDC4 | -1.01 | 1.84E-02 | down |
| MGC45800 | -2.18 | 4.71E-02 | down |
| FAM21A | -1.24 | 3.52E-02 | down |
| CCT6B | -1.29 | 1.02E-02 | down |
| CBLN4 | -1.06 | 4.05E-02 | down |
| NALCN | -1.74 | 1.23E-02 | down |
| DRP2 | -1.33 | 1.04E-02 | down |
| ADAMTSL1 | -1.32 | 2.56E-02 | down |
| INS-IGF2 | -1.1 | 3.64E-02 | down |
| TNNC2 | -1.43 | 4.85E-02 | down |
| HIST1H3G | -1.52 | 3.53E-02 | down |
| HIST1H3E | -1.81 | 2.65E-03 | down |
| PDZRN3 | -1.2 | 2.26E-02 | down |
| CTSG | -1.22 | 1.75E-02 | down |
| SORBS2 | -1.71 | 8.85E-03 | down |
| IL7 | -1.16 | 4.39E-02 | down |
| FMN1 | -1.11 | 3.68E-02 | down |
| C14orf45 | -1.01 | 1.08E-02 | down |
| ZNF238 | -1.35 | 4.60E-03 | down |
| SEMA5A | -1.23 | 2.56E-02 | down |
| FCRL2 | -1.58 | 1.20E-02 | down |
| LRRC6 | -1.29 | 3.15E-02 | down |
| LOC648691 | -1.02 | 1.90E-02 | down |
| FGF14 | -1.82 | 4.06E-03 | down |
| ZFP112 | -1.09 | 1.22E-02 | down |
| BZRAP1 | -1.38 | 4.94E-03 | down |
| C1orf187 | -1.32 | 1.01E-02 | down |
| FANK1 | -1.39 | 2.50E-02 | down |
| RAB37 | -1.15 | 1.61E-02 | down |
| TSNAXIP1 | -1.21 | 2.74E-02 | down |
| RIC3 | -1.6 | 1.68E-02 | down |
| LMO2 | -1.01 | 1.50E-02 | down |
| CCDC147 | -1.14 | 2.78E-02 | down |
| CCDC148 | -1.07 | 1.30E-02 | down |
| MYRIP | -1.51 | 4.77E-02 | down |
| RBM20 | -1.46 | 2.99E-02 | down |
| TCTE1 | -1.5 | 1.88E-02 | down |
| TEKT3 | -1.27 | 4.18E-02 | down |
| TEKT4 | -1.82 | 1.95E-02 | down |
| LOC148709 | -1.56 | 5.51E-03 | down |
| MGC12916 | -1.07 | 4.95E-02 | down |
| CNTN6 | -1.08 | 3.92E-02 | down |
| FLG | -1.47 | 3.02E-02 | down |
| PZP | -1.61 | 3.82E-02 | down |
| RNF183 | -1.72 | 3.27E-02 | down |
| PDZK1 | -1.47 | 4.11E-02 | down |
| SCGB2A2 | -1.84 | 4.14E-02 | down |
| GATA3 | -1.89 | 1.37E-02 | down |
| IL17D | -1.45 | 1.44E-02 | down |
| CPA3 | -2.12 | 2.30E-02 | down |
| SPATA4 | -1.08 | 1.31E-02 | down |
| DNASE1L3 | -1.21 | 1.31E-02 | down |
| LOC442421 | -1.14 | 3.90E-03 | down |
| GABRA5 | -1.21 | 3.21E-02 | down |
| LARGE | -1.02 | 1.82E-02 | down |
| NANOS3 | -1.04 | 3.69E-02 | down |
| SLC6A16 | -1.13 | 4.28E-02 | down |
| IGSF22 | -1.12 | 2.50E-02 | down |
| PYROXD2 | -1.06 | 6.90E-03 | down |
| WFDC3 | -1.36 | 2.92E-02 | down |
| ENOX1 | -1.27 | 3.88E-02 | down |
| CD1E | -1.78 | 1.35E-02 | down |
| CD1A | -1.92 | 2.44E-02 | down |
| ANO4 | -1.59 | 3.08E-02 | down |
| TMEM232 | -1.58 | 2.85E-02 | down |
| ACCN1 | -2.3 | 1.61E-02 | down |
| C14orf50 | -1.12 | 4.45E-02 | down |
| HDC | -1.64 | 4.40E-02 | down |
| CXCR5 | -1.41 | 2.83E-02 | down |
| CD1C | -1.39 | 3.43E-02 | down |
| RLN2 | -1.44 | 1.34E-02 | down |
| C21orf82 | -1.02 | 2.19E-02 | down |
| SLC10A5 | -1.03 | 3.27E-04 | down |
| UPP2 | -1.02 | 2.10E-02 | down |
| C6orf138 | -1.65 | 2.05E-02 | down |
| PIH1D2 | -1.33 | 4.99E-03 | down |
| HTR7 | -1.21 | 9.62E-03 | down |
| PORCN | -1.07 | 3.52E-03 | down |
| EMX2 | -1.68 | 4.48E-02 | down |
| EMX2OS | -1.84 | 2.26E-02 | down |

LogFC: FoldChange
