# Supplementary material for: CKMT1A is a novel potential prognostic biomarker in patients with endometrial cancer
Source: PLoS One. 2022 Jan 25;17(1):e0262000. doi: 10.1371/journal.pone.0262000 (PMC8789190; doi:10.1371/journal.pone.0262000)
Supplement: S4 Table — BP: Biological Process; MF: Molecular Function; CC: Cellular Component; FDR: false discovery rate. (DOC) [file pone.0262000.s004.doc]

**S4 Table..** Results of Gene Set Enrichment Analysis with differentially expressed genes between CKMT1A-high and CKMT1A-low

| BP | GO-term | Description | count in gene set | FDR |
| --- | --- | --- | --- | --- |
|  | GO:0006600 | Creatine metabolic process | 5 of 11 | 2.58E-10 |
|  | GO:0017144 | Drug metabolic process | 8 of 622 | 1.37E-06 |
|  | GO:0006936 | Muscle contraction | 6 of 244 | 2.55E-06 |
|  | GO:0006734 | NADH metabolic process | 4 of 34 | 2.55E-06 |
|  | GO:0044281 | Small molecule metabolic process | 10 of 1779 | 5.26E-06 |
|  | GO:0019752 | Carboxylic acid metabolic process | 8 of 854 | 5.26E-06 |
|  | GO:0006575 | Cellular modified amino acid metabolic process | 5 of 185 | 9.74E-06 |
|  | GO:1901605 | Alpha-amino acid metabolic process | 5 of 209 | 1.61E-05 |
|  | GO:0061718 | Glucose catabolic process to pyruvate | 3 of 25 | 4.29E-05 |
|  | GO:0061621 | Canonical glycolysis | 3 of 25 | 4.29E-05 |
|  | GO:0006735 | NADH regeneration | 3 of 25 | 4.29E-05 |
|  | GO:0006732 | Coenzyme metabolic process | 5 of 297 | 4.80E-05 |
|  | GO:0006757 | ATP generation from ADP | 3 of 39 | 7.46E-05 |
|  | GO:0046434 | Organophosphate catabolic process | 4 of 152 | 8.66E-05 |
|  | GO:0042866 | Pyruvate biosynthetic process | 3 of 42 | 8.66E-05 |
|  | GO:0006094 | Gluconeogenesis | 3 of 46 | 0.0001 |
|  | GO:0009435 | NAD biosynthetic process | 3 of 51 | 0.00013 |
|  | GO:0016310 | Phosphorylation | 7 of 1236 | 0.00019 |
|  | GO:0006090 | Pyruvate metabolic process | 3 of 66 | 0.00021 |
|  | GO:0030388 | Fructose 1,6-bisphosphate metabolic process | 2 of 7 | 0.00022 |
|  | GO:0006754 | ATP biosynthetic process | 3 of 75 | 0.00026 |
|  | GO:0070252 | Actin-mediated cell contraction | 3 of 84 | 0.00035 |
|  | GO:0006796 | Phosphate-containing compound metabolic process | 8 of 2065 | 0.00044 |
|  | GO:0090407 | Organophosphate biosynthetic process | 5 of 577 | 0.00046 |
|  | GO:0009168 | Purine ribonucleoside monophosphate biosynthetic process | 3 of 98 | 0.00048 |
|  | GO:0009166 | Nucleotide catabolic process | 3 of 101 | 0.00051 |
|  | GO:0006000 | Fructose metabolic process | 2 of 15 | 0.00056 |
|  | GO:0046394 | Carboxylic acid biosynthetic process | 4 of 311 | 0.00057 |
|  | GO:0006006 | Glucose metabolic process | 3 of 113 | 0.00061 |
|  | GO:0055086 | Nucleobase-containing small molecule metabolic process | 5 of 662 | 0.0007 |
|  | GO:0019439 | Aromatic compound catabolic process | 4 of 453 | 0.0021 |
|  | GO:0005975 | Carbohydrate metabolic process | 4 of 457 | 0.0021 |
|  | GO:0072521 | Purine-containing compound metabolic process | 4 of 478 | 0.0024 |
|  | GO:0030049 | Muscle filament sliding | 2 of 38 | 0.0025 |
|  | GO:0030029 | Actin filament-based process | 4 of 493 | 0.0025 |
|  | GO:0007015 | Actin filament organization | 3 of 200 | 0.0025 |
|  | GO:0042398 | Cellular modified amino acid biosynthetic process | 2 of 41 | 0.0026 |
|  | GO:0009167 | Purine ribonucleoside monophosphate metabolic process | 3 of 230 | 0.0034 |
|  | GO:0019637 | Organophosphate metabolic process | 5 of 1011 | 0.0035 |
|  | GO:1901135 | Carbohydrate derivative metabolic process | 5 of 1083 | 0.0045 |
|  | GO:1901564 | Organonitrogen compound metabolic process | 10 of 5281 | 0.0068 |
|  | GO:1901566 | Organonitrogen compound biosynthetic process | 5 of 1370 | 0.0122 |
|  | GO:0006941 | Striated muscle contraction | 2 of 111 | 0.0137 |
|  | GO:0055114 | Oxidation-reduction process | 4 of 923 | 0.0175 |
|  | GO:0043312 | Neutrophil degranulation | 3 of 485 | 0.0212 |
|  | GO:0009056 | Catabolic process | 5 of 1859 | 0.0365 |
|  | GO:0009987 | Cellular process | 15 of 14652 | 0.0445 |
| MF | [GO:0004111](http://amigo.geneontology.org/amigo/term/GO:0004111) | Creatine kinase activity | 4 of 6 | 3.92E-09 |
|  | [GO:0004332](http://amigo.geneontology.org/amigo/term/GO:0004332) | Fructose-bisphosphate aldolase activity | 2 of 3 | 0.00015 |
|  | [GO:0008307](http://amigo.geneontology.org/amigo/term/GO:0008307) | Structural constituent of muscle | 2 of 45 | 0.0098 |
|  | [GO:0036094](http://amigo.geneontology.org/amigo/term/GO:0036094) | Small molecule binding | 7 of 2460 | 0.0153 |
|  | [GO:0003824](http://amigo.geneontology.org/amigo/term/GO:0003824) | Catalytic activity | 10 of 5592 | 0.0259 |
|  | [GO:0008092](http://amigo.geneontology.org/amigo/term/GO:0008092) | Cytoskeletal protein binding | 4 of 882 | 0.0292 |
|  | [GO:0005524](http://amigo.geneontology.org/amigo/term/GO:0005524) | ATP binding | 5 of 1462 | 0.0292 |
|  | [GO:0003779](http://amigo.geneontology.org/amigo/term/GO:0003779) | Actin binding | 3 of 413 | 0.0292 |
|  | [GO:0000166](http://amigo.geneontology.org/amigo/term/GO:0000166) | Nucleotide binding | 6 of 2097 | 0.0292 |
|  | [GO:0051015](http://amigo.geneontology.org/amigo/term/GO:0051015) | Actin filament binding | 2 of 158 | 0.0303 |
| CC | [GO:0005862](http://amigo.geneontology.org/amigo/term/GO:0005862) | Muscle thin filament tropomyosin | 2 of 4 | 0.00075 |
|  | [GO:1904813](http://amigo.geneontology.org/amigo/term/GO:1904813) | Ficolin-1-rich granule lumen | 3 of 125 | 0.0054 |
|  | [GO:0044444](http://amigo.geneontology.org/amigo/term/GO:0044444) | Cytoplasmic part | 14 of 9377 | 0.0054 |
|  | [GO:0030017](http://amigo.geneontology.org/amigo/term/GO:0030017) | Sarcomere | 3 of 195 | 0.0054 |
|  | [GO:0005737](http://amigo.geneontology.org/amigo/term/GO:0005737) | Cytoplasm | 15 of 11238 | 0.0054 |
|  | [GO:1904724](http://amigo.geneontology.org/amigo/term/GO:1904724) | Tertiary granule lumen | 2 of 55 | 0.0065 |
|  | [GO:0005884](http://amigo.geneontology.org/amigo/term/GO:0005884) | Actin filament | 2 of 67 | 0.0088 |
|  | [GO:0034774](http://amigo.geneontology.org/amigo/term/GO:0034774) | Secretory granule lumen | 3 of 323 | 0.0118 |
|  | [GO:0015629](http://amigo.geneontology.org/amigo/term/GO:0015629) | Actin cytoskeleton | 3 of 432 | 0.022 |
|  | [GO:0005829](http://amigo.geneontology.org/amigo/term/GO:0005829) | Cytosol | 9 of 4958 | 0.0232 |
|  | [GO:0005743](http://amigo.geneontology.org/amigo/term/GO:0005743) | Mitochondrial inner membrane | 3 of 456 | 0.0232 |
|  | [GO:0005739](http://amigo.geneontology.org/amigo/term/GO:0005739) | Mitochondrion | 5 of 1531 | 0.0232 |
|  | [GO:0044429](http://amigo.geneontology.org/amigo/term/GO:0044429) | Mitochondrial part | 4 of 1015 | 0.0275 |

BP: Biological Process; MF: Molecular Function; CC: Cellular Component; FDR: false discovery rate
