# Supplementary material for: CKMT1A is a novel potential prognostic biomarker in patients with endometrial cancer
Source: PLoS One. 2022 Jan 25;17(1):e0262000. doi: 10.1371/journal.pone.0262000 (PMC8789190; doi:10.1371/journal.pone.0262000)
Supplement: S5 Table — FDR: false discovery rate. (DOC) [file pone.0262000.s005.doc]

**S5 Table.** Results of pathway annotation with differentially expressed genes between CKMT1A-high and CKMT1A-low

| Pathway | Description | Count in gene set | FDR |
| --- | --- | --- | --- |
| hsa00030 | Pentose phosphate pathway | 30 of 120 | 0.00028 |
| hsa00051 | Fructose and mannose metabolism | 33 of 85 | 0.00029 |
| hsa00260 | Glycine, serine and threonine metabolism | 39 of 96 | 0.00035 |
| hsa00330 | Arginine and proline metabolism | 48 of 113 | 1.73E-07 |
| hsa00010 | Glycolysis/Gluconeogenesis | 68 0f 187 | 2.95E-05 |
| hsa01230 | Biosynthesis of amino acids | 72 of 1120 | 2.95E-05 |
| hsa01200 | Carbon metabolism | 116 of 265 | 8.37E-05 |
| hsa01100 | Metabolic pathways | 250 of 1135 | 6.97E-06 |

FDR: false discovery rate
