# Supplementary material for: CKMT1A is a novel potential prognostic biomarker in patients with endometrial cancer
Source: PLoS One. 2022 Jan 25;17(1):e0262000. doi: 10.1371/journal.pone.0262000 (PMC8789190; doi:10.1371/journal.pone.0262000)
Supplement: S6 Table — (DOC) [file pone.0262000.s006.doc]

**S6 Table.** Endometrium sample materials in the Human Protein Atlas

| Endometrium sample ID | Sample description | CKMT1A expression |
| --- | --- | --- |
| GTEX-XV7Q-1526-SM-4BRWB | 40-49 years, female | 2.9 |
| GTEX-U3ZN-0726-SM-4DXT5 | 30-39 years, female | 0.3 |
| GTEX-XUZC-0926-SM-4BOQF | 30-39 years, female | 0.2 |
| GTEX-11EM3-1926-SM-5987U | 20-29 years, female | 0.1 |
| GTEX-13QBU-1626-SM-5LU4S | 40-49 years, female | 0.1 |
| GTEX-PWCY-1426-SM-48TCT | 20-29 years, female | 0.1 |
| GTEX-S32W-1426-SM-4AD66 | 50-59 years, female | 0.1 |
| GTEX-P78B-2526-SM-3P5ZY | 40-49 years, female | 0 |
| GTEX-PWN1-2026-SM-48TD9 | 40-49 years, female | 0 |
| GTEX-PX3G-2026-SM-48U1H | 20-29 years, female | 0 |
| GTEX-R55G-1626-SM-48FF4 | 40-49 years, female | 0 |
| GTEX-T5JW-1526-SM-4DM5E | 20-29 years, female | 0 |
| GTEX-T6MO-1526-SM-4DM57 | 40-49 years, female | 0 |
| GTEX-Y114-1826-SM-4TT87 | 50-59 years, female | 0 |
| GTEX-ZTPG-2026-SM-5O999 | 20-29 years, female | 0 |
| GTEX-ZVT2-0426-SM-5E44S | 50-59 years, female | 0 |
