# Supplementary material for: CKMT1A is a novel potential prognostic biomarker in patients with endometrial cancer
Source: PLoS One. 2022 Jan 25;17(1):e0262000. doi: 10.1371/journal.pone.0262000 (PMC8789190; doi:10.1371/journal.pone.0262000)
Supplement: S7 Table — (DOC) [file pone.0262000.s007.doc]

**S7 Table.** The samples material of endometrial cancer in the Human Protein Atlas

| Sample ID | CKMT expression | Staus | Overall survival (days) |
| --- | --- | --- | --- |
| TCGA-D1-A2G0-01A | 13 | alive | 1484 |
| TCGA-AP-A05J-01A | 11.5 | alive | 1122 |
| TCGA-BS-A0TE-01A | 11 | dead | 146 |
| TCGA-D1-A16G-01A | 9 | dead | 834 |
| TCGA-DF-A2KY-01A | 8.4 | alive | 332 |
| TCGA-AX-A3G7-01A | 7.1 | alive | 9 |
| TCGA-A5-A0GP-01A | 6.8 | alive | 1416 |
| TCGA-PG-A6IB-01A | 6.8 | alive | 648 |
| TCGA-AP-A1DO-01A | 6.5 | alive | 952 |
| TCGA-A5-A0GR-01A | 6.3 | alive | 1120 |
| TCGA-BS-A0UL-01A | 6.2 | alive | 2481 |
| TCGA-AX-A3FX-01A | 6 | dead | 1333 |
| TCGA-B5-A0K4-01A | 6 | alive | 1757 |
| TCGA-AX-A1CE-01A | 5.8 | alive | 1037 |
| TCGA-E6-A1LX-01A | 5.8 | alive | 711 |
| TCGA-EY-A3QX-01A | 5.7 | dead | 989 |
| TCGA-EY-A215-01A | 5.6 | alive | 574 |
| TCGA-AJ-A3I9-01A | 5.5 | dead | 519 |
| TCGA-BS-A0V4-01A | 5.4 | alive | 1810 |
| TCGA-BS-A0TA-01A | 5.2 | dead | 740 |
| TCGA-H5-A2HR-01A | 5.2 | alive | 39 |
| TCGA-AJ-A2QO-01A | 5.1 | dead | 1045 |
| TCGA-EY-A1G8-01A | 5.1 | dead | 456 |
| TCGA-DI-A2QT-01A | 5 | dead | 377 |
| TCGA-BS-A0UV-01A | 5 | alive | 2228 |
| TCGA-AP-A0LH-01A | 4.8 | dead | 1106 |
| TCGA-A5-A0R8-01A | 4.8 | alive | 596 |
| TCGA-AJ-A3BD-01A | 4.6 | alive | 1039 |
| TCGA-D1-A17K-01A | 4.6 | alive | 854 |
| TCGA-AP-A0LT-01A | 4.5 | alive | 1497 |
| TCGA-D1-A2G6-01A | 4.1 | dead | 258 |
| TCGA-DF-A2KS-01A | 4.1 | alive | 3318 |
| TCGA-AX-A2IO-01A | 4.1 | alive | 1975 |
| TCGA-D1-A17H-01A | 4 | alive | 852 |
| TCGA-D1-A1O8-01A | 3.9 | dead | 120 |
| TCGA-A5-A7WK-01A | 3.9 | alive | 560 |
| TCGA-B5-A11N-01A | 3.9 | alive | 1876 |
| TCGA-AP-A05H-01A | 3.9 | alive | 1385 |
| TCGA-AP-A056-01A | 3.9 | alive | 2618 |
| TCGA-AP-A0LO-01A | 3.8 | dead | 1016 |
| TCGA-EY-A1GM-01A | 3.7 | dead | 995 |
| TCGA-EO-A3KX-01A | 3.7 | alive | 1170 |
| TCGA-A5-A0GV-01A | 3.7 | alive | 1643 |
| TCGA-D1-A16S-01A | 3.7 | alive | 568 |
| TCGA-FI-A2EX-01A | 3.6 | alive | 284 |
| TCGA-FI-A2D4-01A | 3.5 | dead | 243 |
| TCGA-AP-A5FX-01A | 3.5 | dead | 1099 |
| TCGA-AX-A05Y-01A | 3.5 | alive | 2684 |
| TCGA-BG-A0RY-01A | 3.5 | alive | 469 |
| TCGA-AP-A054-01A | 3.4 | dead | 709 |
| TCGA-EO-A2CH-01A | 3.4 | dead | 590 |
| TCGA-A5-A0GB-01A | 3.4 | alive | 997 |
| TCGA-D1-A1O7-01A | 3.4 | alive | 32 |
| TCGA-BG-A0W1-01A | 3.4 | alive | 1607 |
| TCGA-BG-A221-01A | 3.2 | alive | 55 |
| TCGA-BS-A0U9-01B | 3.1 | alive | 2825 |
| TCGA-EY-A549-01A | 3.1 | alive | 605 |
| TCGA-DF-A2L0-01A | 3 | dead | 788 |
| TCGA-B5-A0K9-01A | 3 | dead | 409 |
| TCGA-A5-A7WJ-01A | 3 | alive | 341 |
| TCGA-B5-A11J-01A | 3 | alive | 1656 |
| TCGA-EY-A2ON-01A | 2.9 | dead | 610 |
| TCGA-DI-A1NO-01A | 2.9 | dead | 285 |
| TCGA-A5-A0GM-01A | 2.9 | alive | 1841 |
| TCGA-B5-A1MX-01A | 2.9 | alive | 1473 |
| TCGA-AX-A05W-01A | 2.9 | alive | 2450 |
| TCGA-BG-A222-01A | 2.9 | alive | 291 |
| TCGA-AX-A1CF-01A | 2.9 | alive | 2337 |
| TCGA-BS-A0T9-01A | 2.8 | dead | 1428 |
| TCGA-EY-A54A-01A | 2.8 | alive | 447 |
| TCGA-AX-A2IN-01A | 2.7 | dead | 947 |
| TCGA-BS-A0V8-01A | 2.7 | alive | 2544 |
| TCGA-KP-A3W1-01A | 2.7 | alive | 288 |
| TCGA-AX-A3G9-01A | 2.7 | alive | 558 |
| TCGA-EO-A22T-01A | 2.6 | alive | 2035 |
| TCGA-BG-A0MH-01A | 2.6 | alive | 1930 |
| TCGA-EO-A22S-01A | 2.6 | alive | 1872 |
| TCGA-K6-A3WQ-01A | 2.5 | dead | 427 |
| TCGA-EY-A1GC-01A | 2.5 | alive | 1647 |
| TCGA-QF-A5YS-01A | 2.5 | alive | 689 |
| TCGA-D1-A160-01A | 2.5 | alive | 112 |
| TCGA-EO-A3B0-01A | 2.5 | alive | 1412 |
| TCGA-E6-A1LZ-01A | 2.5 | alive | 2406 |
| TCGA-AX-A1C7-01A | 2.5 | alive | 1054 |
| TCGA-B5-A11R-01A | 2.3 | alive | 2349 |
| TCGA-AX-A06F-01A | 2.3 | alive | 1748 |
| TCGA-AJ-A3EM-01A | 2.3 | alive | 677 |
| TCGA-A5-A2K7-01A | 2.3 | alive | 1063 |
| TCGA-A5-A0RA-01A | 2.3 | alive | 884 |
| TCGA-AP-A05A-01A | 2.2 | dead | 1944 |
| TCGA-DI-A2QU-01A | 2.2 | dead | 946 |
| TCGA-D1-A1O0-01A | 2.2 | alive | 63 |
| TCGA-AX-A2HG-01A | 2.2 | alive | 1163 |
| TCGA-AX-A3G6-01A | 2.2 | alive | 8 |
| TCGA-EY-A72D-01A | 2.2 | alive | 179 |
| TCGA-D1-A174-01A | 2.2 | alive | 594 |
| TCGA-B5-A3FA-01A | 2.1 | alive | 2929 |
| TCGA-AP-A3K1-01A | 2 | dead | 666 |
| TCGA-A5-A0G1-01A | 2 | dead | 3251 |
| TCGA-D1-A3JP-01A | 2 | alive | 515 |
| TCGA-AX-A0IZ-01A | 2 | alive | 1333 |
| TCGA-AP-A05D-01A | 2 | alive | 1758 |
| TCGA-AX-A06B-01A | 2 | alive | 2058 |
| TCGA-AX-A1CI-01A | 2 | alive | 2600 |
| TCGA-DF-A2KN-01A | 2 | alive | 67 |
| TCGA-EY-A2OO-01A | 2 | alive | 1392 |
| TCGA-B5-A11E-01A | 2 | alive | 1820 |
| TCGA-EY-A1GQ-01A | 2 | alive | 544 |
| TCGA-FI-A2EY-01A | 1.9 | dead | 880 |
| TCGA-AX-A2H4-01A | 1.9 | dead | 916 |
| TCGA-EY-A5W2-01A | 1.9 | alive | 516 |
| TCGA-DF-A2KR-01A | 1.9 | alive | 2178 |
| TCGA-D1-A15X-01A | 1.9 | alive | 909 |
| TCGA-D1-A1NX-01A | 1.9 | alive | 555 |
| TCGA-AX-A3G4-01A | 1.9 | alive | 9 |
| TCGA-A5-A3LO-01A | 1.9 | alive | 776 |
| TCGA-BG-A0MQ-01A | 1.9 | alive | 1817 |
| TCGA-D1-A0ZV-01A | 1.9 | alive | 728 |
| TCGA-AP-A0LD-01A | 1.9 | alive | 3589 |
| TCGA-D1-A169-01A | 1.9 | alive | 1135 |
| TCGA-EY-A1GV-01A | 1.9 | alive | 481 |
| TCGA-A5-A0G9-01A | 1.9 | alive | 2540 |
| TCGA-A5-A0VO-01A | 1.8 | dead | 875 |
| TCGA-AJ-A3OL-01A | 1.8 | dead | 449 |
| TCGA-AX-A2HD-01A | 1.8 | alive | 1414 |
| TCGA-B5-A3FC-01A | 1.8 | alive | 2185 |
| TCGA-AX-A3G8-01A | 1.8 | alive | 30 |
| TCGA-AJ-A3BG-01A | 1.8 | alive | 550 |
| TCGA-BG-A0MU-01A | 1.8 | alive | 617 |
| TCGA-B5-A1MV-01A | 1.7 | alive | 1565 |
| TCGA-AX-A3G3-01A | 1.7 | alive | 42 |
| TCGA-AP-A1DM-01A | 1.7 | alive | 1700 |
| TCGA-AX-A3FW-01A | 1.7 | alive | 17 |
| TCGA-DF-A2KV-01A | 1.7 | alive | 2717 |
| TCGA-BG-A0M8-01A | 1.7 | alive | 2020 |
| TCGA-EO-A3AZ-01A | 1.7 | alive | 826 |
| TCGA-AX-A063-01A | 1.7 | alive | 2417 |
| TCGA-BG-A0MG-01A | 1.7 | alive | 1477 |
| TCGA-D1-A16F-01A | 1.7 | alive | 80 |
| TCGA-AJ-A5DW-01A | 1.7 | alive | 390 |
| TCGA-D1-A17U-01A | 1.7 | alive | 697 |
| TCGA-AP-A059-01A | 1.7 | alive | 1461 |
| TCGA-D1-A3DG-01A | 1.6 | dead | 130 |
| TCGA-A5-A1OG-01A | 1.6 | dead | 973 |
| TCGA-DI-A1NN-01A | 1.6 | alive | 1076 |
| TCGA-BK-A13C-01A | 1.6 | alive | 760 |
| TCGA-AX-A1CA-01A | 1.6 | alive | 974 |
| TCGA-BG-A0YV-01A | 1.6 | alive | 382 |
| TCGA-EO-A3AS-01A | 1.6 | alive | 1046 |
| TCGA-B5-A11H-01A | 1.6 | alive | 2687 |
| TCGA-DI-A0WH-01A | 1.6 | alive | 496 |
| TCGA-B5-A11G-01A | 1.6 | alive | 3448 |
| TCGA-D1-A16E-01A | 1.6 | alive | 1119 |
| TCGA-AJ-A3BF-01A | 1.6 | alive | 551 |
| TCGA-EY-A3L3-01A | 1.6 | alive | 832 |
| TCGA-AX-A1C5-01A | 1.6 | alive | 1001 |
| TCGA-EY-A548-01A | 1.6 | alive | 430 |
| TCGA-AX-A062-01A | 1.6 | alive | 1399 |
| TCGA-AP-A05O-01A | 1.6 | alive | 384 |
| TCGA-A5-A0GD-01A | 1.5 | dead | 3423 |
| TCGA-QS-A8F1-01A | 1.5 | dead | 135 |
| TCGA-EY-A1GS-01A | 1.5 | dead | 670 |
| TCGA-AX-A3FT-01A | 1.5 | alive | 6 |
| TCGA-AJ-A2QM-01A | 1.5 | alive | 894 |
| TCGA-AX-A3GI-01A | 1.5 | alive | 12 |
| TCGA-D1-A0ZS-01A | 1.5 | alive | 148 |
| TCGA-B5-A0JZ-01A | 1.5 | alive | 2173 |
| TCGA-AX-A05S-01A | 1.4 | dead | 413 |
| TCGA-EY-A1GX-01A | 1.4 | dead | 1127 |
| TCGA-EC-A1QX-01A | 1.4 | alive | 224 |
| TCGA-EY-A1H0-01A | 1.4 | alive | 588 |
| TCGA-EO-A3AU-01A | 1.4 | alive | 1543 |
| TCGA-A5-A0GH-01A | 1.4 | alive | 2254 |
| TCGA-BS-A0TI-01A | 1.4 | alive | 1882 |
| TCGA-AX-A06J-01A | 1.4 | alive | 913 |
| TCGA-BG-A0MC-01A | 1.4 | alive | 1263 |
| TCGA-B5-A0K8-01A | 1.3 | alive | 847 |
| TCGA-EY-A1GL-01A | 1.3 | alive | 1014 |
| TCGA-A5-A0R7-01A | 1.3 | alive | 535 |
| TCGA-D1-A0ZQ-01A | 1.3 | alive | 260 |
| TCGA-AP-A0L9-01A | 1.3 | alive | 3815 |
| TCGA-D1-A16D-01A | 1.3 | alive | 1141 |
| TCGA-B5-A5OD-01A | 1.3 | alive | 1038 |
| TCGA-AJ-A3TW-01A | 1.3 | alive | 513 |
| TCGA-EO-A3KU-01A | 1.3 | alive | 677 |
| TCGA-AX-A1C4-01A | 1.3 | alive | 404 |
| TCGA-EY-A214-01A | 1.3 | alive | 438 |
| TCGA-BG-A0VW-01A | 1.3 | alive | 1582 |
| TCGA-BS-A0UM-01A | 1.3 | alive | 2644 |
| TCGA-D1-A1NW-01A | 1.3 | alive | 22 |
| TCGA-AX-A2HC-01A | 1.3 | alive | 1044 |
| TCGA-B5-A11Y-01A | 1.3 | alive | 560 |
| TCGA-AP-A1DH-01A | 1.2 | alive | 2180 |
| TCGA-AJ-A8CV-01A | 1.2 | alive | 1068 |
| TCGA-EO-A3AV-01A | 1.2 | alive | 1069 |
| TCGA-AX-A2HA-01A | 1.2 | alive | 2475 |
| TCGA-D1-A1NZ-01A | 1.2 | alive | 548 |
| TCGA-EY-A1GH-01A | 1.2 | alive | 710 |
| TCGA-D1-A17L-01A | 1.2 | alive | 802 |
| TCGA-AP-A0LP-01A | 1.2 | alive | 1875 |
| TCGA-AX-A06H-01A | 1.2 | alive | 833 |
| TCGA-KP-A3W3-01A | 1.2 | alive | 794 |
| TCGA-EO-A22X-01A | 1.2 | alive | 1752 |
| TCGA-AJ-A5DV-01A | 1.2 | alive | 679 |
| TCGA-FI-A2F4-01A | 1.2 | alive | 2230 |
| TCGA-B5-A11L-01B | 1.1 | dead | 2240 |
| TCGA-A5-A2K4-01A | 1.1 | dead | 871 |
| TCGA-KJ-A3U4-01A | 1.1 | alive | 1095 |
| TCGA-AP-A051-01A | 1.1 | alive | 3948 |
| TCGA-PG-A7D5-01A | 1.1 | alive | 1177 |
| TCGA-AJ-A2QK-01A | 1.1 | alive | 643 |
| TCGA-EO-A1Y5-01A | 1.1 | alive | 327 |
| TCGA-AJ-A3NG-01A | 1.1 | alive | 475 |
| TCGA-D1-A16J-01A | 1.1 | alive | 910 |
| TCGA-B5-A11Z-01A | 1.1 | alive | 528 |
| TCGA-BG-A0LX-01A | 1.1 | alive | 614 |
| TCGA-BS-A0U7-01A | 1 | dead | 935 |
| TCGA-BG-A3PP-01A | 1 | alive | 681 |
| TCGA-EY-A1GP-01A | 1 | alive | 573 |
| TCGA-AJ-A3BI-01A | 1 | alive | 575 |
| TCGA-A5-A0GW-01A | 1 | alive | 706 |
| TCGA-BS-A0TD-01A | 1 | alive | 2379 |
| TCGA-EO-A3AY-01A | 1 | alive | 1158 |
| TCGA-A5-A0GI-01A | 1 | alive | 2682 |
| TCGA-A5-AB3J-01A | 1 | alive | 512 |
| TCGA-AX-A06L-01A | 1 | alive | 1845 |
| TCGA-EO-A1Y7-01A | 1 | alive | 1495 |
| TCGA-D1-A0ZO-01A | 1 | alive | 602 |
| TCGA-B5-A0JV-01A | 1 | alive | 2616 |
| TCGA-D1-A1NS-01A | 1 | alive | 43 |
| TCGA-KP-A3VZ-01A | 1 | alive | 722 |
| TCGA-BS-A0UT-01A | 1 | alive | 2212 |
| TCGA-D1-A177-01A | 1 | alive | 548 |
| TCGA-D1-A0ZP-01A | 1 | alive | 911 |
| TCGA-B5-A5OE-01A | 1 | alive | 1063 |
| TCGA-AJ-A3NE-01A | 1 | alive | 628 |
| TCGA-EY-A212-01A | 0.9 | dead | 310 |
| TCGA-BG-A0MI-01A | 0.9 | dead | 714 |
| TCGA-A5-A1OH-01A | 0.9 | alive | 1105 |
| TCGA-BK-A0C9-01A | 0.9 | alive | 1092 |
| TCGA-EO-A22Y-01A | 0.9 | alive | 1567 |
| TCGA-BS-A0U5-01A | 0.9 | alive | 2935 |
| TCGA-D1-A17F-01A | 0.9 | alive | 39 |
| TCGA-EY-A1GF-01A | 0.9 | alive | 826 |
| TCGA-EO-A3KW-01A | 0.9 | alive | 497 |
| TCGA-EO-A1Y8-01A | 0.9 | alive | 1147 |
| TCGA-AX-A0IW-01A | 0.9 | alive | 1149 |
| TCGA-A5-A1OK-01A | 0.9 | alive | 917 |
| TCGA-SL-A6JA-01A | 0.9 | alive | 7 |
| TCGA-B5-A0JN-01A | 0.9 | alive | 2445 |
| TCGA-B5-A0JU-01B | 0.9 | alive | 1802 |
| TCGA-AX-A0IS-01A | 0.8 | dead | 1858 |
| TCGA-BG-A0MA-01A | 0.8 | dead | 326 |
| TCGA-AX-A3G1-01A | 0.8 | dead | 624 |
| TCGA-FI-A3PX-01A | 0.8 | alive | 2384 |
| TCGA-BS-A0V6-01A | 0.8 | alive | 2725 |
| TCGA-D1-A101-01A | 0.8 | alive | 53 |
| TCGA-AX-A1C8-01A | 0.8 | alive | 776 |
| TCGA-AX-A060-01A | 0.8 | alive | 1075 |
| TCGA-B5-A11V-01A | 0.8 | alive | 595 |
| TCGA-AX-A05T-01A | 0.8 | alive | 1010 |
| TCGA-BS-A0TG-01A | 0.8 | alive | 2371 |
| TCGA-E6-A1M0-01A | 0.8 | alive | 1793 |
| TCGA-A5-A2K2-01A | 0.8 | alive | 3595 |
| TCGA-AX-A1CJ-01A | 0.8 | alive | 2233 |
| TCGA-QS-A5YR-01A | 0.8 | alive | 719 |
| TCGA-BS-A0V7-01A | 0.8 | alive | 1934 |
| TCGA-B5-A0JY-01A | 0.8 | alive | 2107 |
| TCGA-B5-A3FB-01A | 0.8 | alive | 2844 |
| TCGA-D1-A17M-01A | 0.8 | alive | 311 |
| TCGA-D1-A0ZU-01A | 0.8 | alive | 872 |
| TCGA-BG-A0YU-01A | 0.8 | alive | 451 |
| TCGA-AP-A05N-01A | 0.7 | dead | 726 |
| TCGA-FI-A3PV-01A | 0.7 | dead | 1249 |
| TCGA-B5-A0K3-01A | 0.7 | dead | 830 |
| TCGA-B5-A1MS-01B | 0.7 | dead | 3112 |
| TCGA-E6-A2P8-01A | 0.7 | dead | 72 |
| TCGA-B5-A1MY-01A | 0.7 | dead | 361 |
| TCGA-FI-A2F8-01A | 0.7 | alive | 933 |
| TCGA-A5-A0GQ-01A | 0.7 | alive | 2247 |
| TCGA-D1-A163-01A | 0.7 | alive | 991 |
| TCGA-D1-A1NU-01A | 0.7 | alive | 402 |
| TCGA-B5-A11X-01A | 0.7 | alive | 596 |
| TCGA-BG-A0M3-01A | 0.7 | alive | 1071 |
| TCGA-QF-A5YT-01A | 0.7 | alive | 1215 |
| TCGA-B5-A11O-01A | 0.7 | alive | 2609 |
| TCGA-AJ-A3NC-01A | 0.7 | alive | 1125 |
| TCGA-PG-A916-01A | 0.7 | alive | 522 |
| TCGA-A5-A3LP-01A | 0.7 | alive | 455 |
| TCGA-B5-A0K6-01A | 0.7 | alive | 1953 |
| TCGA-D1-A16X-01A | 0.7 | alive | 111 |
| TCGA-B5-A3S1-01A | 0.7 | alive | 902 |
| TCGA-BK-A4ZD-01A | 0.7 | alive | 506 |
| TCGA-EY-A210-01A | 0.7 | alive | 383 |
| TCGA-EY-A1GI-01A | 0.7 | alive | 710 |
| TCGA-D1-A162-01A | 0.7 | alive | 1145 |
| TCGA-BG-A0M9-01A | 0.7 | alive | 2270 |
| TCGA-BG-A18C-01A | 0.7 | alive | 470 |
| TCGA-BG-A0M7-01A | 0.7 | alive | 1937 |
| TCGA-AX-A1C9-01A | 0.7 | alive | 802 |
| TCGA-BK-A0CA-01A | 0.7 | alive | 1064 |
| TCGA-B5-A11Q-01A | 0.7 | alive | 976 |
| TCGA-EY-A1GU-01A | 0.6 | dead | 997 |
| TCGA-B5-A1N2-01A | 0.6 | dead | 954 |
| TCGA-AX-A2H7-01A | 0.6 | dead | 1241 |
| TCGA-D1-A17R-01A | 0.6 | dead | 339 |
| TCGA-DI-A2QY-01A | 0.6 | dead | 3349 |
| TCGA-BG-A220-01A | 0.6 | alive | 434 |
| TCGA-D1-A161-01A | 0.6 | alive | 892 |
| TCGA-D1-A15W-01A | 0.6 | alive | 915 |
| TCGA-SJ-A6ZI-01A | 0.6 | alive | 631 |
| TCGA-AP-A0LM-01A | 0.6 | alive | 825 |
| TCGA-BG-A0MS-01A | 0.6 | alive | 1882 |
| TCGA-BG-A0MO-01A | 0.6 | alive | 1309 |
| TCGA-BG-A0VT-01A | 0.6 | alive | 1568 |
| TCGA-D1-A103-01A | 0.6 | alive | 669 |
| TCGA-BG-A2AD-01A | 0.6 | alive | 609 |
| TCGA-D1-A3JQ-01A | 0.6 | alive | 526 |
| TCGA-AJ-A3OJ-01A | 0.6 | alive | 467 |
| TCGA-AP-A0LJ-01A | 0.6 | alive | 1421 |
| TCGA-B5-A3F9-01A | 0.6 | alive | 4155 |
| TCGA-KP-A3W0-01A | 0.6 | alive | 404 |
| TCGA-BS-A0U8-01A | 0.6 | alive | 2963 |
| TCGA-EY-A1GK-01A | 0.6 | alive | 693 |
| TCGA-D1-A16Y-01A | 0.6 | alive | 532 |
| TCGA-A5-A0GX-01A | 0.6 | alive | 2088 |
| TCGA-AP-A0LV-01A | 0.6 | alive | 763 |
| TCGA-BG-A3EW-01A | 0.6 | alive | 344 |
| TCGA-AP-A052-01A | 0.5 | dead | 669 |
| TCGA-AX-A2H2-01A | 0.5 | dead | 145 |
| TCGA-BG-A0MK-01A | 0.5 | dead | 1317 |
| TCGA-AJ-A3EJ-01A | 0.5 | dead | 50 |
| TCGA-AJ-A23M-01A | 0.5 | dead | 616 |
| TCGA-D1-A16I-01A | 0.5 | dead | 350 |
| TCGA-AP-A053-01A | 0.5 | dead | 3365 |
| TCGA-B5-A0K0-01A | 0.5 | alive | 1478 |
| TCGA-AJ-A3NF-01A | 0.5 | alive | 645 |
| TCGA-BK-A0CB-01A | 0.5 | alive | 1092 |
| TCGA-BG-A0VV-01A | 0.5 | alive | 1553 |
| TCGA-AJ-A3BK-01A | 0.5 | alive | 1031 |
| TCGA-QS-A5YQ-01A | 0.5 | alive | 759 |
| TCGA-BG-A18A-01A | 0.5 | alive | 426 |
| TCGA-AP-A0LG-01A | 0.5 | alive | 2015 |
| TCGA-BK-A26L-01C | 0.5 | alive | 734 |
| TCGA-BS-A0UA-01A | 0.5 | alive | 3495 |
| TCGA-AP-A0LE-01A | 0.5 | alive | 3357 |
| TCGA-EO-A2CG-01A | 0.5 | alive | 1564 |
| TCGA-A5-A2K5-01A | 0.5 | alive | 752 |
| TCGA-A5-A1OF-01A | 0.5 | alive | 973 |
| TCGA-D1-A0ZR-01A | 0.5 | alive | 17 |
| TCGA-D1-A17T-01A | 0.5 | alive | 71 |
| TCGA-BG-A186-01A | 0.5 | alive | 419 |
| TCGA-DF-A2KU-01A | 0.5 | alive | 3067 |
| TCGA-A5-A0G3-01A | 0.5 | alive | 1428 |
| TCGA-D1-A175-01A | 0.5 | alive | 202 |
| TCGA-A5-A1OJ-01A | 0.5 | alive | 1006 |
| TCGA-AX-A05Z-01A | 0.5 | alive | 2175 |
| TCGA-AX-A1CN-01A | 0.5 | alive | 1143 |
| TCGA-2E-A9G8-01A | 0.5 | alive | 1249 |
| TCGA-B5-A1MZ-01A | 0.5 | alive | 1598 |
| TCGA-AJ-A3QS-01A | 0.5 | alive | 513 |
| TCGA-EO-A3L0-01A | 0.5 | alive | 1554 |
| TCGA-AP-A0LI-01A | 0.5 | alive | 2972 |
| TCGA-5B-A90C-01A | 0.5 | alive | 1202 |
| TCGA-D1-A2G7-01A | 0.4 | dead | 1793 |
| TCGA-AP-A1DP-01A | 0.4 | dead | 1081 |
| TCGA-FI-A2D2-01A | 0.4 | dead | 2352 |
| TCGA-QS-A744-01A | 0.4 | alive | 451 |
| TCGA-B5-A11P-01B | 0.4 | alive | 1583 |
| TCGA-B5-A0JT-01A | 0.4 | alive | 1682 |
| TCGA-B5-A5OC-01A | 0.4 | alive | 1249 |
| TCGA-D1-A16Q-01A | 0.4 | alive | 324 |
| TCGA-BG-A0LW-01A | 0.4 | alive | 566 |
| TCGA-D1-A3DA-01A | 0.4 | alive | 483 |
| TCGA-FI-A2EW-01A | 0.4 | alive | 1462 |
| TCGA-BS-A0VI-01A | 0.4 | alive | 2646 |
| TCGA-BS-A0TC-01A | 0.4 | alive | 2602 |
| TCGA-D1-A16B-01A | 0.4 | alive | 1106 |
| TCGA-B5-A0JS-01A | 0.4 | alive | 2627 |
| TCGA-E6-A2P9-01A | 0.4 | alive | 1386 |
| TCGA-FI-A2D0-01A | 0.4 | alive | 1023 |
| TCGA-BK-A56F-01A | 0.4 | alive | 401 |
| TCGA-AJ-A8CW-01A | 0.4 | alive | 4 |
| TCGA-D1-A176-01A | 0.4 | alive | 917 |
| TCGA-D1-A2G5-01A | 0.4 | alive | 1455 |
| TCGA-BG-A0M2-01A | 0.4 | alive | 637 |
| TCGA-BG-A0M6-01A | 0.3 | dead | 671 |
| TCGA-D1-A15Z-01A | 0.3 | dead | 58 |
| TCGA-BK-A139-01C | 0.3 | dead | 943 |
| TCGA-D1-A179-01A | 0.3 | dead | 448 |
| TCGA-JU-AAVI-01A | 0.3 | dead | 540 |
| TCGA-B5-A1MU-01A | 0.3 | dead | 1548 |
| TCGA-A5-A0GA-01A | 0.3 | dead | 543 |
| TCGA-AX-A064-01A | 0.3 | alive | 1517 |
| TCGA-SL-A6J9-01A | 0.3 | alive | 2 |
| TCGA-B5-A0K7-01A | 0.3 | alive | 1933 |
| TCGA-AX-A3GB-01A | 0.3 | alive | 440 |
| TCGA-BK-A6W4-01A | 0.3 | alive | 300 |
| TCGA-B5-A121-01A | 0.3 | alive | 504 |
| TCGA-B5-A0JR-01A | 0.3 | alive | 1731 |
| TCGA-B5-A0K2-01A | 0.3 | alive | 2209 |
| TCGA-B5-A1MW-01A | 0.3 | alive | 447 |
| TCGA-EC-A1NJ-01A | 0.3 | alive | 488 |
| TCGA-D1-A0ZZ-01A | 0.3 | alive | 686 |
| TCGA-D1-A17S-01A | 0.3 | alive | 739 |
| TCGA-AX-A0IU-01A | 0.3 | alive | 1087 |
| TCGA-AX-A1CK-01A | 0.3 | alive | 2441 |
| TCGA-D1-A17N-01A | 0.3 | alive | 46 |
| TCGA-PG-A5BC-01A | 0.3 | alive | 707 |
| TCGA-AP-A05P-01A | 0.3 | alive | 636 |
| TCGA-A5-A2K3-01A | 0.3 | alive | 2012 |
| TCGA-B5-A11F-01A | 0.3 | alive | 5651 |
| TCGA-BG-A18B-01A | 0.3 | alive | 470 |
| TCGA-AJ-A23N-01A | 0.2 | dead | 439 |
| TCGA-AP-A1DQ-01A | 0.2 | dead | 1423 |
| TCGA-D1-A16V-01A | 0.2 | dead | 120 |
| TCGA-B5-A3FD-01A | 0.2 | dead | 1197 |
| TCGA-AX-A06D-01A | 0.2 | dead | 373 |
| TCGA-FI-A2D6-01A | 0.2 | dead | 767 |
| TCGA-AJ-A3NH-01A | 0.2 | dead | 310 |
| TCGA-AX-A0J0-01A | 0.2 | alive | 1220 |
| TCGA-BG-A0VX-01A | 0.2 | alive | 1043 |
| TCGA-AP-A1DV-01A | 0.2 | alive | 204 |
| TCGA-EY-A2OQ-01A | 0.2 | alive | 885 |
| TCGA-AJ-A8CT-01A | 0.2 | alive | 82 |
| TCGA-B5-A11I-01A | 0.2 | alive | 2303 |
| TCGA-AP-A0LL-01A | 0.2 | alive | 2554 |
| TCGA-BG-A2AE-01A | 0.2 | alive | 721 |
| TCGA-AJ-A2QL-01A | 0.2 | alive | 602 |
| TCGA-AJ-A3IA-01A | 0.2 | alive | 633 |
| TCGA-DI-A1BY-01A | 0.2 | alive | 1114 |
| TCGA-BG-A0M0-01A | 0.2 | alive | 588 |
| TCGA-DI-A1BU-01A | 0.2 | alive | 1195 |
| TCGA-AJ-A3OK-01A | 0.2 | alive | 485 |
| TCGA-EY-A1GE-01A | 0.2 | alive | 741 |
| TCGA-AX-A2HH-01A | 0.2 | alive | 540 |
| TCGA-D1-A3DH-01A | 0.2 | alive | 573 |
| TCGA-EY-A1GO-01A | 0.2 | alive | 586 |
| TCGA-D1-A17B-01A | 0.2 | alive | 213 |
| TCGA-A5-A0VQ-01A | 0.2 | alive | 485 |
| TCGA-PG-A915-01A | 0.2 | alive | 249 |
| TCGA-AJ-A2QN-01A | 0.2 | alive | 1069 |
| TCGA-AP-A1E1-01A | 0.2 | alive | 1395 |
| TCGA-AX-A1CP-01A | 0.2 | alive | 1107 |
| TCGA-AW-A1PO-01A | 0.2 | alive | 17 |
| TCGA-D1-A17C-01A | 0.2 | alive | 30 |
| TCGA-BG-A0VZ-01A | 0.2 | alive | 1601 |
| TCGA-B5-A11M-01A | 0.2 | alive | 1362 |
| TCGA-BG-A0W2-01A | 0.2 | alive | 1721 |
| TCGA-D1-A17Q-01A | 0.2 | alive | 54 |
| TCGA-AX-A2HJ-01A | 0.2 | alive | 935 |
| TCGA-AP-A0LN-01A | 0.2 | alive | 2510 |
| TCGA-EY-A1GD-01A | 0.2 | alive | 1639 |
| TCGA-5S-A9Q8-01A | 0.2 | alive | 313 |
| TCGA-EO-A22U-01A | 0.2 | alive | 1407 |
| TCGA-BG-A0M4-01A | 0.2 | alive | 2167 |
| TCGA-BK-A6W3-01A | 0.2 | alive | 305 |
| TCGA-A5-A0GG-01A | 0.2 | alive | 3762 |
| TCGA-AX-A2HK-01A | 0.1 | dead | 90 |
| TCGA-EY-A1G7-01A | 0.1 | dead | 189 |
| TCGA-EY-A4KR-01A | 0.1 | dead | 346 |
| TCGA-AP-A0L8-01A | 0.1 | dead | 1484 |
| TCGA-AJ-A23O-01A | 0.1 | dead | 312 |
| TCGA-BK-A0CC-01A | 0.1 | alive | 1067 |
| TCGA-A5-A0R9-01A | 0.1 | alive | 749 |
| TCGA-D1-A16R-01A | 0.1 | alive | 21 |
| TCGA-BS-A0WQ-01A | 0.1 | alive | 2022 |
| TCGA-D1-A15V-01A | 0.1 | alive | 1201 |
| TCGA-D1-A16O-01A | 0.1 | alive | 1023 |
| TCGA-D1-A0ZN-01A | 0.1 | alive | 512 |
| TCGA-D1-A1O5-01A | 0.1 | alive | 458 |
| TCGA-D1-A16N-01A | 0.1 | alive | 945 |
| TCGA-PG-A917-01A | 0.1 | alive | 212 |
| TCGA-FI-A2CX-01A | 0.1 | alive | 2031 |
| TCGA-AX-A2H5-01A | 0.1 | alive | 2167 |
| TCGA-D1-A167-01A | 0.1 | alive | 478 |
| TCGA-EY-A1GT-01A | 0.1 | alive | 490 |
| TCGA-AX-A1CC-01A | 0.1 | alive | 788 |
| TCGA-A5-A0G5-01A | 0.1 | alive | 1126 |
| TCGA-AX-A2H8-01A | 0.1 | alive | 1861 |
| TCGA-AX-A1CR-01A | 0.1 | alive | 701 |
| TCGA-D1-A102-01A | 0.1 | alive | 135 |
| TCGA-B5-A0JX-01A | 0.1 | alive | 1787 |
| TCGA-AP-A1DR-01A | 0.1 | alive | 1495 |
| TCGA-D1-A165-01A | 0.1 | alive | 20 |
| TCGA-AP-A0LF-01A | 0.1 | alive | 2859 |
| TCGA-A5-A0GU-01A | 0.1 | alive | 860 |
| TCGA-AP-A1E4-01A | 0.1 | alive | 1555 |
| TCGA-A5-A0R6-01A | 0.1 | alive | 1153 |
| TCGA-FI-A2CY-01A | 0.1 | alive | 2080 |
| TCGA-EC-A24G-01A | 0.1 | alive | 511 |
| TCGA-B5-A0K1-01A | 0.1 | alive | 1110 |
| TCGA-FI-A2EU-01A | 0.1 | alive | 2941 |
| TCGA-AX-A3FS-01A | 0.1 | alive | 1 |
| TCGA-AP-A1DK-01A | 0.1 | alive | 2767 |
| TCGA-B5-A11W-01A | 0.1 | alive | 593 |
| TCGA-AP-A1E0-01A | 0.1 | alive | 1832 |
| TCGA-BS-A0UJ-01A | 0.1 | alive | 2506 |
| TCGA-B5-A11U-01A | 0.1 | alive | 509 |
| TCGA-BS-A0UF-01A | 0.1 | alive | 2611 |
| TCGA-BG-A187-01A | 0.1 | alive | 448 |
| TCGA-D1-A17D-01A | 0.1 | alive | 926 |
| TCGA-EY-A547-01A | 0.1 | alive | 545 |
| TCGA-EY-A1GR-01A | 0.1 | alive | 536 |
| TCGA-B5-A3FH-01A | 0.1 | alive | 841 |
| TCGA-AP-A0LS-01A | 0.1 | alive | 2904 |
| TCGA-EY-A1GW-01A | 0 | dead | 337 |
| TCGA-KP-A3W4-01A | 0 | dead | 554 |
| TCGA-FI-A2D5-01A | 0 | dead | 421 |
| TCGA-B5-A11S-01A | 0 | alive | 2229 |
| TCGA-BK-A13B-01A | 0 | alive | 966 |
| TCGA-A5-A0GE-01A | 0 | alive | 3205 |
| TCGA-BG-A2L7-01A | 0 | alive | 612 |
| TCGA-D1-A168-01A | 0 | alive | 1098 |
| TCGA-EO-A22R-01A | 0 | alive | 1878 |
| TCGA-FI-A2F9-01A | 0 | alive | 1803 |
| TCGA-EY-A2OM-01A | 0 | alive | 694 |
| TCGA-DF-A2KZ-01A | 0 | alive | 826 |
| TCGA-AJ-A3EL-01A | 0 | alive | 684 |
| TCGA-A5-A0G2-01A | 0 | alive | 4549 |
| TCGA-AX-A2HF-01A | 0 | alive | 848 |
| TCGA-PG-A914-01A | 0 | alive | 687 |
| TCGA-AJ-A3EK-01A | 0 | alive | 446 |
| TCGA-A5-A0GJ-01A | 0 | alive | 2000 |
| TCGA-BG-A0MT-01A | 0 | alive | 644 |
| TCGA-SJ-A6ZJ-01A | 0 | alive | 556 |
| TCGA-E6-A8L9-01A | 0 | alive | 1013 |
| TCGA-4E-A92E-01A | 0 | alive | 13 |
| TCGA-B5-A0KB-01B | 0 | alive | 824 |
| TCGA-EO-A3B1-01A | 0 | alive | 820 |
| TCGA-AP-A1E3-01A | 0 | alive | 847 |
| TCGA-AX-A05U-01A | 0 | alive | 903 |
| TCGA-AX-A3FZ-01A | 0 | alive | 2 |
| TCGA-A5-A0VP-01A | 0 | alive | 1288 |
| TCGA-B5-A1MR-01A | 0 | alive | 6859 |
